# Reactions O(^3^P, ^1^D) + HCCCN(X^1^Σ^+^) (Cyanoacetylene): Crossed-Beam and Theoretical Studies and Implications for the Chemistry of Extraterrestrial Environments

**DOI:** 10.1021/acs.jpca.2c07708

**Published:** 2023-01-13

**Authors:** Pengxiao Liang, Emilia V. F. de Aragão, Giacomo Pannacci, Gianmarco Vanuzzo, Andrea Giustini, Demian Marchione, Pedro Recio, Francesco Ferlin, Domenico Stranges, Noelia Faginas Lago, Marzio Rosi, Piergiorgio Casavecchia, Nadia Balucani

**Affiliations:** † Dipartimento di Chimica, Biologia e Biotecnologie, Università degli Studi di Perugia, Perugia 06123, Italy; ‡ Master-Tec srl, Via Sicilia 41, Perugia 06128, Italy; § Dipartimento di Chimica, Università degli Studi La Sapienza, Roma 00185, Italy; ∥ Dipartimento di Ingegneria Civile e Ambientale, Università degli Studi di Perugia, Perugia 06123, Italy

## Abstract

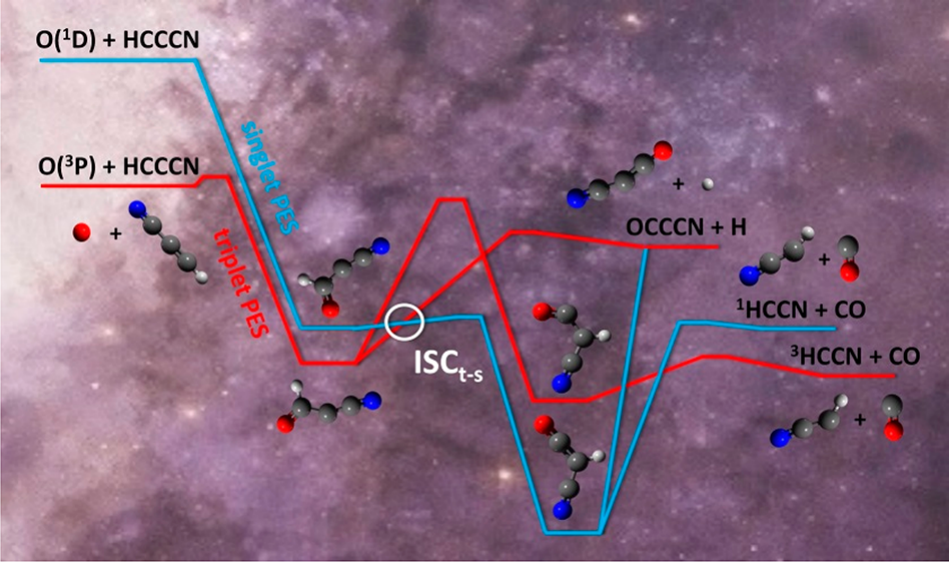

Cyanoacetylene **(**HCCCN), the first member of the cyanopolyyne family (HC_*n*_N, where *n* = 3, 5, 7, ...), is of particular interest in astrochemistry being ubiquitous in space (molecular clouds, solar-type protostars, protoplanetary disks, circumstellar envelopes, and external galaxies) and also relatively abundant. It is also abundant in the upper atmosphere of Titan and comets. Since oxygen is the third most abundant element in space, after hydrogen and helium, the reaction O + HCCCN can be of relevance in the chemistry of extraterrestrial environments. Despite that, scarce information exists not only on the reactions of oxygen atoms with cyanoacetylene but with nitriles in general. Here, we report on a combined experimental and theoretical investigation of the reactions of cyanoacetylene with both ground ^3^P and excited ^1^D atomic oxygen and provide detailed information on the primary reaction products, their branching fractions (BFs), and the overall reaction mechanisms. More specifically, the reactions of O(^3^P, ^1^D) with HCCCN(X^1^Σ^+^) have been investigated under single-collision conditions by the crossed molecular beams scattering method with mass spectrometric detection and time-of-flight analysis at the collision energy, *E*_c_, of 31.1 kJ/mol. From product angular and time-of-flight distributions, we have identified the primary reaction products and determined their branching fractions (BFs). Theoretical calculations of the relevant triplet and singlet potential energy surfaces (PESs) were performed to assist the interpretation of the experimental results and clarify the reaction mechanism. Adiabatic statistical calculations of product BFs for the decomposition of the main triplet and singlet intermediates have also been carried out. Merging together the experimental and theoretical results, we conclude that the O(^3^P) reaction is characterized by a minor adiabatic channel leading to OCCCN (cyanoketyl) + H (experimental BF = 0.10 ± 0.05), while the dominant channel (BF = 0.90 ± 0.05) occurs via intersystem crossing to the underlying singlet PES and leads to formation of ^1^HCCN (cyanomethylene) + CO. The O(^1^D) reaction is characterized by the same two channels, with the relative CO/H yield being slightly larger. Considering the recorded reactive signal and the calculated entrance barrier, we estimate that the rate coefficient for reaction O(^3^P) + HC_3_N at 300 K is in the 10^–12^ cm^3^ molec^–1^ s^–1^ range. Our results are expected to be useful to improve astrochemical and photochemical models. In addition, they are also relevant in combustion chemistry, because the thermal decomposition of pyrrolic and pyridinic structures present in fuel-bound nitrogen generates many nitrogen-bearing compounds, including cyanoacetylene.

## 1 Introduction

Oxygen is an important player in the chemistry of the universe, being the third most abundant element. Even though its mole fraction is only 477 ppm, it is more abundant than carbon (326 ppm) and nitrogen (102 ppm) and exhibits a rich chemistry, contrarily to the two most abundant elements, hydrogen and helium (mole fraction of 90.9964% and 8.8714%, respectively). In cold objects of the interstellar medium (ISM), it is assumed to be largely depleted from the gas phase being the main constituent of the water ice mantles that cover interstellar dust particles, while a significant fraction is also segregated into CO, a very abundant interstellar molecule. However, residual atomic oxygen in its ground electronic state, O(^3^P_2,1,0_), is still present in a large amount, also in cold regions (see refs (1, 2), and references therein), and can have a strong impact both in the formation and in the destruction of interstellar complex organic molecules.^3,4^ In particular, because of its capability of reacting with organic molecules in a destructive way, the presence of atomic oxygen can severely reduce the chemical complexity of the available organic species.

Over the past several years, we have shown numerous cases in which O(^3^P) degrades organic molecules. Specifically, we have analyzed several examples of reactions of O(^3^P) with unsaturated hydrocarbons: acetylene,^5^ ethylene,^6−8^ propene,^9,10^ propyne,^11,12^ allene,^13^ 1-butene,^14^ 1,2-butadiene,^15^ 1,3-butadiene,^16^ and, more recently, also small aromatic compounds (benzene^17,18^ and pyridine^19^). We have seen that oxygen atoms are even more effective than we thought in inducing the breakup of C–C bonds and in degrading the hydrocarbons directly toward CO or CO precursors because of intersystem crossing (ISC) to the underlying singlet potential energy surface (PES).^6−19^ However, at the same time, the reactions of O(^3^P) with organic molecules allow for the formation of other complex molecular species^4,20^ that can, in turn, foster the chemical growth toward complexity. All those processes, indeed, can form also new O-containing organic molecules (e.g., ketene, phenol, butenone) or O-containing radicals that can further react, leading to the formation of other O-rich organic molecules. Some of them (glycolaldehyde, acetic acid) are widely detected in space and are considered to be prebiotic species, being potential precursors of sugars and amino acids. Quite interestingly, indeed, among the so-called interstellar complex organic molecules (iCOMs), those which are by far the most abundant do contain oxygen, namely, methanol, dimethyl ether, methyl formate, etc.^21,22^

In the present study, we extend the same combined experimental and theoretical approach to the reaction of O(^3^P) with a particularly relevant interstellar molecule, the ubiquitous cyanoacetylene (HCCCN). Interstellar HC_3_N was first detected in 1971 at 9.0977 GHz (*J* = 2–1) in the galactic star-forming region Sgr B2^23^ and has since been observed in a variety of interstellar environments, including molecular clouds, solar-type protostars, circumstellar envelopes, and external galaxies.^23−31^ It is also one of the few molecules observed in protoplanetary disks (GO Tau, MWC 480, and LkCa 15)^32^ and it has been detected in cometary comae (C/1995 O1 Hale-Bopp,^33^ 67P/Churyumov-Gerasimenko,^34^ C/2014 Q2 Lovejoy^35^) and in the upper atmosphere of Titan, the massive moon of Saturn.^36^ In addition to being ubiquitous, interstellar HC_3_N has a relatively large abundance with respect to H_2_ ranging between 10^–11^ and 10^–8^ in different sources.^26^ Cyanoacetylene is also the simplest member of the cyanopolyyne family (HC_*n*_N, where *n* = 3, 5, 7, etc.) widely abundant in star-forming regions. Since we already know that O(^3^P) degrades acetylene, of which HC_3_N is a derivative, the copresence of both species in some regions of the ISM and in comets might imply that the title reaction contributes to control the abundance of HC_3_N or other cyanopolyynes.

In addition, atomic oxygen in its first electronically excited state, O(^1^D), has been clearly detected in cometary comae where it is produced by the photodissociation of several parent species such as H_2_O or CO/CO_2_.^37^ Given the low number density of the comae, O(^1^D) mainly decays by spontaneous emission (the radiative lifetime is ca. 110 s). Its emission is actually used as a tracer of water molecules, because atomic oxygen in the excited state ^1^D can be formed only chemically since the transition from the ground-state O(^3^P) is forbidden. However, it is worth mentioning here that oxygen atoms in the ^1^D state are incredibly reactive with closed shell species and bimolecular reactions have been recently called into play to explain the formation of molecules detected in cometary comae.^38^ Another reason for being interested in the reactions of O(^1^D) with interstellar molecules is associated with the recent suggestion that its reactions with molecules present in interstellar ice can lead to iCOMs.^39^ By producing O(^1^D) via the photodissociation of solid O_2_ or CO_2_ at λ < 200 nm, the formation of methanol and formaldehyde in the presence of CH_4_ ice was observed, as well as ethanol and acetaldehyde in the presence of solid C_2_H_6_, ethylene oxide and acetaldehyde in the presence of solid C_2_H_4_, and ketene in the presence of solid C_2_H_2_. In other words, the reactions of O(^1^D) with organic molecules present in interstellar ices could contribute to the formation of oxygenated organic molecules with some loss of hydrogen on the icy surface of interstellar grains.^39^

In addition to its astrochemical relevance, the reaction of O(^3^P) with cyanoacetylene is also of importance in combustion chemistry. In fact, some of the most dangerous air pollutants are the well-known nitrogen oxides NO_*x*_.^40^ The major anthropogenic source of NO_*x*_ is the combustion of heavy fuels, like coals and coal-derived liquids: they contain a large amount of nitrogen in pyrrolic and pyridinic structures,^41−43^ and their decomposition at high temperature produces NO_*x*_ precursors, among which cyanoacetylene and cyanoethylene (C_2_H_3_CN) are abundant.^44−47^ Therefore, the study of their subsequent reactions with oxygen atoms, always present in combustion environments, is central to unveil the NO_*x*_ evolution for those fuels.

Despite the relevance of the O(^3^P) + HC_3_N reaction in both astrophysical and combustion environments, there have been very few experimental/theoretical studies on this reaction. In 2001, Borget et al.^48^ investigated the reaction of HC_3_N with atomic oxygen generated from photodissociation of ozone (O_3_) at 255 nm on a water ice surface at 7 K. They observed and characterized the formation of cyanoketene (CNCHCO). This species corresponds to the most stable intermediate on the ground-state singlet PES and can be formed by the barrierless (on water ice surface) O(^3^P) addition on the triple C≡C bond of HC_3_N, followed by intersystem crossing to the ground-state singlet PES and H migration and then by collisional stabilization. Alternatively, the dominant O(^1^D) species produced by the 255 nm photolysis of O_3_ can directly add to the triple C≡C bond, leading, after ready 1–2 H shift, to singlet cyanoketene, which is then stabilized by the surface.

In 2006, Xie et al.^49^ theoretically studied the reaction mechanism of oxygen atoms with HC_3_N, both in the gas phase and on water ices. Both triplet and singlet PESs were determined using different methods/levels of theory, and the possibility of ISC was considered, although no detailed theoretical treatment of ISC was pursued. The reaction was determined to exhibit a substantial (from about 15 to 21 kJ/mol, depending on the theory level) entrance barrier on the triplet PES in the gas phase, but the same reaction was found to be barrierless when occurring on the water ice surface at 7 K. It was concluded that in the gas phase, among the possible exothermic product channels on the triplet PES (the energies of the various reaction channels are at the Gaussian-3 level from ref (49)),
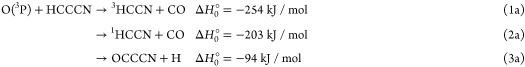
the most exothermic adiabatic channel (1a) leading to ground-state ^3^HCCN (cyanomethylene, also termed cyanocarbene) + CO is the most important one, with the channel (3a) leading (adiabatically) to OCCCN (cyanoketyl) + H being minor. The possibility of ISC around the minimum of the initial triplet diradical intermediate was also considered (but not quantified theoretically) for the O(^3^P) reaction, and this could lead to the spin-forbidden ^1^HCCN + CO product channel (2a).

Also, the reaction of O(^1^D) with HC_3_N, relative to the same three most exothermic channels,
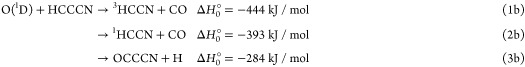
was envisaged to lead dominantly to ^3^HCCN + CO formation (channel 1b) via efficient ISC from the singlet to the triplet PES in the exit channel. None of the above suggestions could be verified at that time, because experimental information on the product identity of the O(^3^P, ^1^D) + HC_3_N reactions and on their branching fractions (BFs) was not available in the literature.

In this paper, given the lack of experimental data in the gas phase on the title reactions and considering the uncertainty associated with the product branching fractions (an important piece of information for astrochemical and photochemical models), we have conducted a combined experimental and theoretical investigation on the reaction of O(^3^P) with HC_3_N(X^1^Σ^+^) using the crossed molecular beam (CMB) scattering technique with mass spectrometric (MS) detection and electronic structure calculations to elucidate the primary product(s), their BFs, and relative formation pathway(s). The goal is to provide useful information for inclusion in improved astrochemical, photochemical, and combustion models as the reaction O(^3^P) + HC_3_N is not considered to date even though similar reactions (such as O(^3^P) + C_2_H_2_, O(^3^P) + C_2_H_4_, and O(^3^P) + CH_3_CCH) have been considered by Occhiogrosso et al.^3^ and by Harada et al.^50^ to model warm temperature (*T*) interstellar regions. In addition, due to the presence of some O(^1^D) in our atomic oxygen beam, also information on the O(^1^D) + HC_3_N reaction dynamics is provided. The experimental results are discussed in the light of dedicated electronic calculations of the triplet/singlet C_3_HON PESs and statistical Rice–Ramsperger–Kassel–Marcus/master equation (RRKM/ME) calculations of product BFs on adiabatic triplet and singlet PESs. In contrast to the previous theoretical suggestions, it is found that the O(^3^P) reaction dynamics/kinetics with HC_3_N is dominated by ISC from the entrance triplet PES to the underlying singlet PES, leading to the spin-forbidden ^1^HCCN + CO product channel (BF = 0.90 ± 0.05), while the H-displacement channel, produced adiabatically on the triplet PES, is minor yet substantial (BF = 0.10 ± 0.05). Comparisons of the derived reaction dynamics, product BFs, and extent of ISC with those of the related O(^3^P) + HCC–CH_3_ (propyne) reaction are carried out. The reaction O(^1^D) + HC_3_N is found to lead to the same two product channels, with the ^1^HCCN + CO channel being comparatively slightly larger (BF = 0.94 ± 0.03) than in the O(^3^P) reaction. The entrance barrier of the O(^3^P) reaction is theoretically found to be significantly lower than previously predicted, which makes the title reaction more relevant than thought in the cold extraterrestrial environments.

HC_3_N is a molecule with a recognized prebiotic potential (as many unsaturated nitriles), and therefore, within the framework of the Italian National Project of Astrobiology,^51^ we have recently investigated its reactions with other reactive radicals that are abundant in extraterrestrial environments where HC_3_N has been identified, such as N(^2^D)^52^ (Titan and comets) and CN^53−55^ (also unpublished results) (Titan, interstellar clouds, and comets). This work is providing another piece in the puzzle of cyanoacetylene chemistry in space.

The paper is structured as follows. In sections 2 and 3, we describe the experimental and theoretical methods, respectively. Section 4 will report the experimental results and their analysis, while section 5 will describe the triplet and singlet PESs and the results of the statistical calculations of product BFs. The combined experimental/theoretical findings will then be discussed in section 6, while the implications for the chemistry of extraterrestrial as well as combustion environments will be commented on in section 7. The key points of the present study will be summarized in the concluding section 8.

## 2 Experimental Method

The dynamics of the O(^3^P) + HCCCN reaction was investigated using the CMB technique with a rotatable quadrupole mass-spectrometer (MS) detector and TOF analysis system. The basis of the method and details of the CMB apparatus have been described elsewhere.^56−63^ Briefly, two supersonic beams of the reactants are crossed at an angle of 90° inside a large scattering chamber kept at a base pressure of 2 × 10^–7^ hPa (operating pressure about 1 × 10^–6^ hPa). The reaction products scattered from the collision region enter a triply differentially pumped, ultrahigh vacuum chamber, in the inner region of which the ionization takes place by an electron-impact ionizer, featuring tunable electron energy; the ions are then selected by a quadrupole mass filter and collected by a Daly type detector.^64^ The detector angular resolution for a point collision zone is 1.1°. The “single-collision conditions” of the experiment allow the unambiguous identification of the primary reaction products, because the nascent products formed at the collision region reach the detector before undergoing collisions with any other molecule or walls.

The supersonic oxygen beam was generated using a radio frequency (RF) discharge beam source^65−67^ in which 85 hPa of a diluted O_2_(5%)/He gas mixture was discharged at 300 W of RF power, through a 0.48 mm diameter water-cooled quartz nozzle followed by a 0.8 mm diameter boron nitride skimmer and a further collimating aperture. The resulting beam is characterized by a predominance (≥90%) of atomic oxygen in its ground electronic state (^3^P), with a small fraction (≤10%) of atomic oxygen in its first electronically excited state (^1^D).^65^ The O(^3^P, ^1^D) beam has a peak velocity of 2162 m/s and a speed ratio of 4.4.

The cyanoacetylene (HC_3_N) molecular beam was generated by expanding 67 hPa of the neat species through a stainless nozzle of 0.1 mm diameter. The HC_3_N beam has a peak velocity of 657 m/s and a speed ratio of 3.5, as in our previous study of the HC_3_N reaction with N(^2^D).^52^ The resulting collision energy, *E*_c_, is 31.1 kJ/mol and the center-of-mass angle, Θ_CM_, 44.1°. Notably, because cyanoacetylene is not easily commercially available, for this study, it was synthesized before usage following the two-stage method described in the literature^68^ and reported in ref (52).

The product angular distribution *N*(Θ), namely, the intensity of the products as a function of the laboratory (LAB) scattering angle Θ, is recorded by the MS detector that can rotate in the collision plane, around the axis orthogonal to the plane containing the crossing reagent beams. During the *N*(Θ) measurements, the HC_3_N molecular beam is modulated at 160 Hz by a tuning fork chopper for background subtraction. Product TOF distributions, *N*(Θ, *t*), are obtained at selected LAB angles employing the TOF pseudorandom chopping technique based on a pseudorandom wheel containing four identical sequences of 127 open/closed elements, spinning in front of the entrance of the detector at 328.1 Hz (corresponding to a dwell time of 6 μs/channel).

For a quantitative and physical interpretation of the scattering event and to achieve a detailed understanding of the reaction dynamics, it is necessary to move from the LAB reference frame to the center-of-mass (CM) frame.^57−63^ The CM flux, *I*_CM_(θ, *u*), of the products is related to the LAB product number density, *N*_LAB_(Θ), through the following equation: *N*_LAB_(Θ, *v*) = *I*_CM_(θ, *u*) (where *v* and *u* are the velocity in the LAB and in the CM frame, respectively, and the term  is the transformation Jacobian).^58^ Because of the finite resolution of the experimental conditions, *I*_CM_(θ, *u*), or rather *I*_CM_(θ, *E*_*T*_^′^) (where *E*_*T*_^′^ is the translational energy), which can be factorized into the product of the angular (*T*(θ)) and translational energy (*P*(*E*_*T*_^′^)) distributions, is derived by a forward convolution fit of the total product LAB angular and TOF distributions at a given mass to charge (*m*/*z*) ratio^59,60^ according to the relation
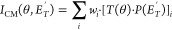
with the parameter *w*_*i*_ representing the relative contribution of the integral cross section of the *i*th channel.^59^

## 3 Computational Methods

### 3.1 Electronic Structure Calculations

The potential energy surfaces for the O(^3^P, ^1^D) + HC_3_N system have been investigated through the optimization of the most stable stationary points along the reactive pathways. Following an established computational scheme already described in previous studies,^69−75^ minima and saddle point geometries were optimized using density functional theory (DFT), with the Becke, three-parameter, Lee–Yang–Parr (B3LYP) functional,^76,77^ in conjunction with the correlation consistent valence polarized basis set aug-cc-pVTZ.^78^ At the same level of theory, vibrational frequency analysis was performed to obtain the zero-point energy correction at 0 K and confirm the nature of each stationary point, i.e., a minimum, if all frequencies are real, and a saddle point, if just one imaginary frequency is present. Likewise, at the same level of theory, intrinsic reaction coordinate (IRC)^79,80^ calculations were performed in order to confirm that each saddle point is connected to the corresponding optimized intermediates of the PES. At last, for each stationary point, a single-point calculation was performed by employing the coupled-cluster CCSD(T)^81−83^ method in conjunction with the same basis set. All calculations were performed by adopting an unrestricted formalism using the Gaussian 09 code.^84^

In order to obtain higher accuracy of the calculated energies (minima, maxima, and products) of the most relevant pathways, we decided to compute them at a higher level of calculation, using the same approach recently employed for the O(^3^P) + 1,3-butadiene reaction^16^ where a complete basis set extrapolation and a correction for the core–valence correlation were considered. In this approach, the energy is computed as
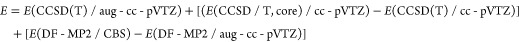
where, using Martin’s two-parameter scheme for extrapolation,^85^
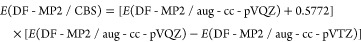
MOLPRO was used for these calculations.^86^

### 3.2 RRKM Calculations

In order to investigate the active unimolecular pathways of the PES, we implemented a kinetic model solving the one-dimensional master equation through the usage of the MultiWell program package provided by Barker et al.^87−89^ RRKM microcanonical rate coefficients *k*(*E*) of each channel were determined as a function of energy *E* on the basis of harmonic frequencies using the conventional transition state theory (TST) for *tight* transition states, where counts of sums and densities of states were carried out by employing the DenSum subprogram as implemented in MultiWell.^87−89^ For barrierless reactions, such as the H loss on the singlet surface and for the bimolecular entrance channels, the variational transition state theory (VTST) was adopted by performing B3LYP/aug-cc-pVTZ constrained optimizations at fixed distances between the two interacting species, followed by an analysis of the harmonic vibrational frequencies orthogonal to the reaction coordinate. Energies of each optimized geometry were subsequently refined at the CCSD(T)/aug-cc-pVTZ level. In this regard, the subprogram Ktools of the MultiWell program package^87−89^ has been used to calculate microcanonical rates for *loose* transition states.

## 4 Experimental Results and Analysis

According to the previous^49^ and present electronic structure calculations, for the O(^3^P) + HC_3_N reaction, there are five possible exothermic channels, one nearly thermoneutral, while several others are substantially endothermic:
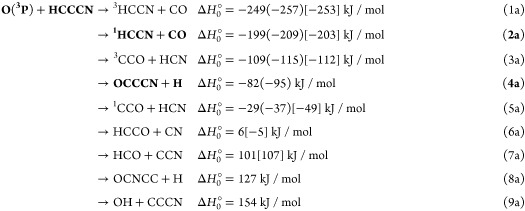
The reported standard enthalpies of reaction at 0 K, Δ*H*_0_^°^, are those calculated in the present work at the CCSD(T) level and at the CCSD(T)/CBS level for channels 1–3 (values in parentheses). In square brackets are the values from available enthalpies of formation at 0 K.^90−92^ As can be seen there is good agreement between the experimental data (when available) and the most accurate theoretical evaluation.

In our CMB experiments, we have been able to detect the reactive signal associated with channels 2a and 4a (highlighted in bold). Of the above channels, 2a can only be formed via ISC from the triplet to the underlying singlet PES in the entrance channel of the reaction. While channels 1a and 3a can occur only adiabatically on the triplet PES, channels 4a and 6a can occur on both the triplet and singlet PESs. On the other hand, channel 5a, although exothermic, cannot be formed via ISC from the triplet to the singlet PES, due to unfavorable kinetics. Channels 7a–9a are energetically closed at the experimental *E*_c_. We have probed all six channels (1a–6a).

For the reaction of O(^1^D), all of the above nine channels are exothermic:
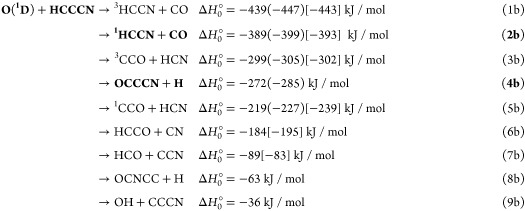
However, as we will see further below, only the bolded channels (2b and 4b) are those occurring in the O(^1^D) reaction under our experimental conditions.

### 4.1 LAB Product Angular and TOF Distributions

**Figure 1:**
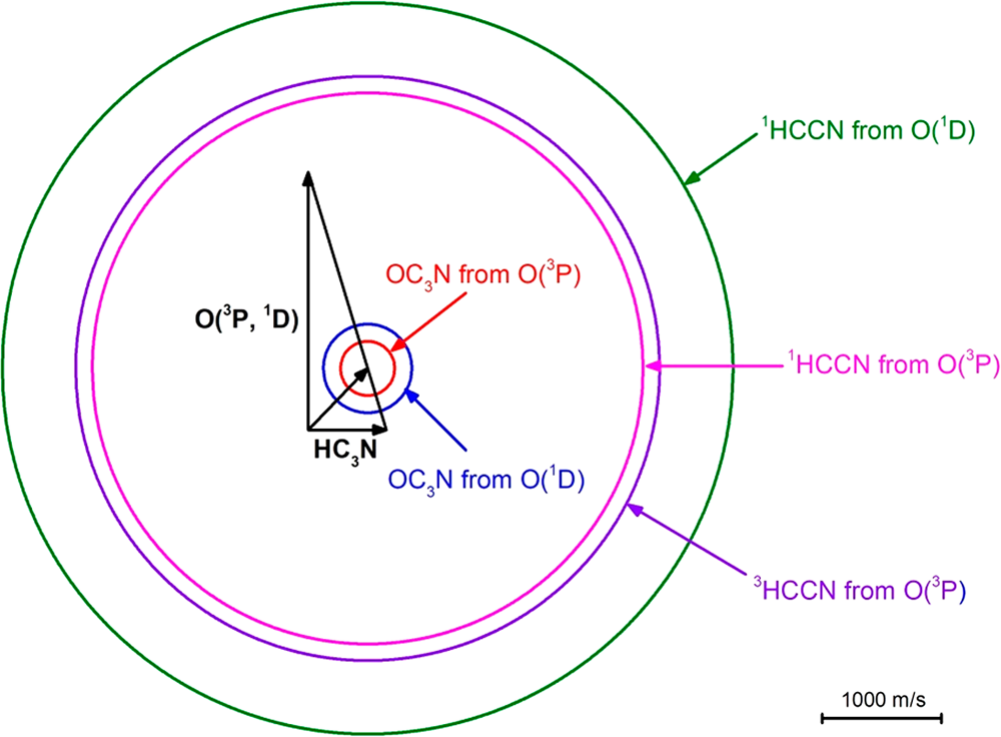
Newton (velocity vector) diagram of the experiment. Here the various circles delimit the maximum speed that the indicated products from the O(^3^P, ^1^D) + HC_3_N reactions at *E*_c_ = 31.1 kJ/mol can attain if all the available energy is channeled into product translational energy. Red line: Newton circles for the H-displacement channel that leads to OC_3_N from the O(^3^P) reaction. Blue line: same for the O(^1^D) reaction. Magenta line: Newton circle for the ^1^HCCN product from the O(^3^P) reaction (via ISC). Violet line: Newton circle for the ^3^HCCN product from the adiabatic O(^3^P) reaction. Green line: Newton circle for the ^1^HCCN product from the adiabatic O(^1^D) reaction.

Before presenting the experimental results, it is useful to illustrate the velocity vector (so-called “Newton”) diagram of the system which depicts the kinematics of the O(^3^P, ^1^D) + HC_3_N reactions at *E*_c_ = 31.1 kJ/mol and how the different possible products can be scattered in angle and velocity with respect to the center-of-mass of the system. The most probable Newton diagram for the O(^3^P, ^1^D) + HC_3_N reactions is depicted in Figure 1, where the superimposed circles are drawn by considering the maximum CM speed that each (indicated) product can attain if all the total available energy, *E*_TOT_, for that channel (*E*_TOT_ = *E*_c_ – Δ*H*_0_^°^) is converted into product translational energy. Only the experimentally observed channels from the O(^3^P) and O(^1^D) reactions are depicted. It can be easily appreciated that the H-displacement channels 4a and 4b are those with the most favorable kinematics. The detected OC_3_N heavy coproducts are confined within much smaller Newton circles (and, therefore, strongly enhanced in the LAB frame by a favorable CM → LAB Jacobian transformation^58^) compared to those associated with the products (HCCN) detected for the C–C bond-breaking channels 1a/2a and 1b/2b. In these cases, two cofragments of comparable mass are produced and, because of linear momentum conservation,^57−60^ the HCCN products are scattered over a much wider Newton circle.

Experimentally, reactive scattering signals were observed and then measured at *m*/*z* = 66 (OCCCN^+^) and *m*/*z* = 38 (CCN^+^). *Hard* (70 eV) electron ionization was initially used for data collection. However, it was soon noted that for *m*/*z* = 38 there were some interferences originating from daughter ions of the cyanoacetylene reactant elastically/inelastically scattered by the oxygen beam. To mitigate and essentially suppress this interfering signal, we resorted to *soft* ionization (28 eV electrons was sufficient) at *m*/*z* = 38. Then, also the distributions at *m*/*z* = 66 were measured at 28 eV for normalization purposes. The relative intensities (*m*/*z* = 66)/(*m*/*z* = 38) were 0.7/1.0 at Θ = 44°, using soft ionization at 28 eV.

While *m*/*z* = 66 corresponds to the parent ion of the heavy coproduct, OC_3_N, of channel 4a and possibly also 4b, *m*/*z* = 38 (CCN^+^) corresponds to the (−1) daughter ion of the HCCN product from channels 1a, 1b, 2a, and 2b and possibly also to the (−28) daughter ion of the OC_3_N product from channels 4a and 4b. HCCN products were detected at the daughter ion *m*/*z* = 38 because the neutral HCCN (*m*/*z* = 39) strongly fragments to CCN^+^ in the ionizer, even upon soft ionization at 28 eV, and the background at *m*/*z* = 38 in our MS detector was about 1 order of magnitude lower than that at *m*/*z* = 39. In previous studies of HCCN formation from the reaction *N*(^2^D) + C_2_H_2_, we measured a ratio (*m*/*z* = 38)/(*m*/*z* = 39) of about 1.6 at 70 eV electron energy.^93^ We have assumed this same ratio to hold also at 28 eV. We remark that, although the fragmentation of HCCN at 28 eV is expected to be somewhat lower than that at 70 eV, if for instance we assume a ratio of unity (rather than 1.6) in the data analysis, the variation in the derived values of the product BFs falls within the overall uncertainty (≈30–50%) of the determinations.

Detection of the HCCO and CN coproducts of channels 6a and 6b was attempted at their parent masses *m*/*z* = 41 and 26, respectively, but no reactive signal was observed within our sensitivity, which suggests a negligible contribution to the reaction from the nearly thermoneutral channel (6a) and also from the exothermic channel (6b). We have not found a reactive signal at *m*/*z* = 40 (CCO) or 27 (HCN), and we conclude that channels 3a and 5a from O(^3^P) and channels 3b and 5b from O(^1^D) are also negligible. We can then reasonably assume that also the less exothermic channels 7b, 8b, and 9b from O(^1^D) are negligible at *E*_c_ = 31.1 kJ/mol.

### 4.2 The *m*/*z* = 66 Data: H-Displacement Channels

**Figure 2:**
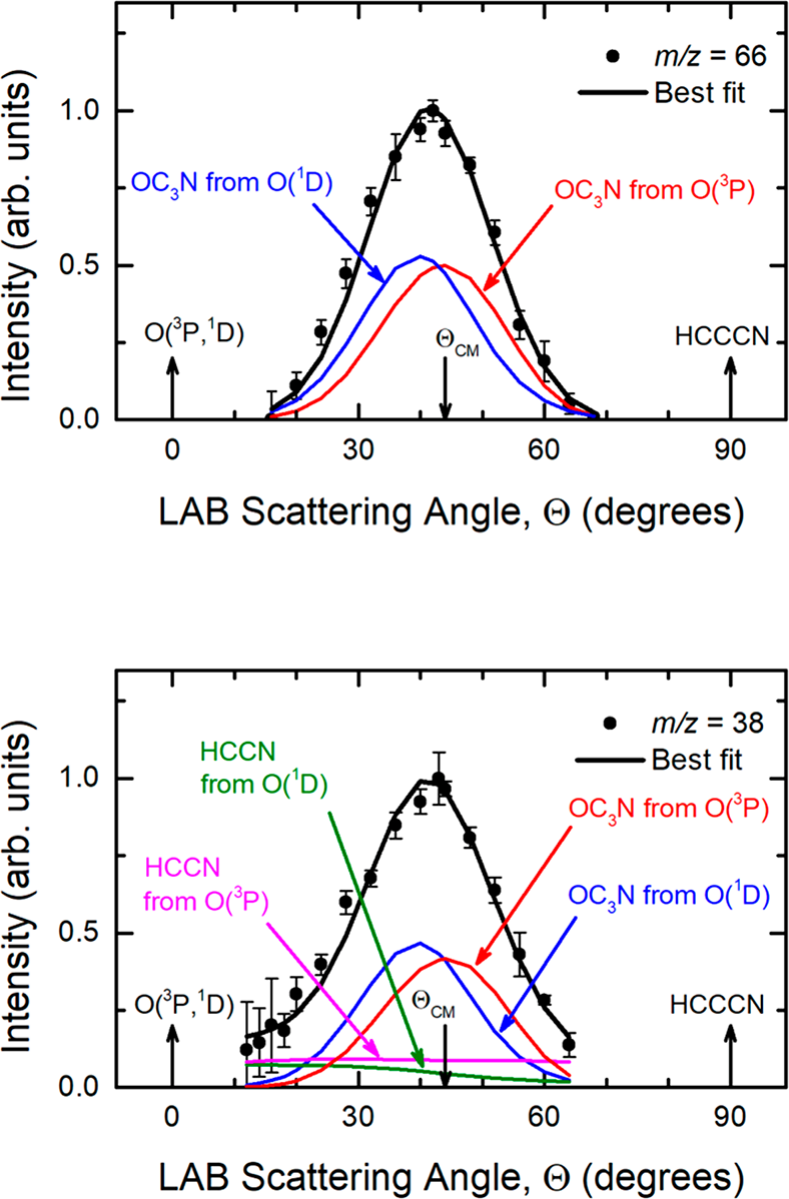
LAB angular distributions measured at *m*/*z* = 66 (top panel) and *m*/*z* = 38 (bottom panel) for the O(^3^P, ^1^D) + HC_3_N reactions at *E*_c_ = 31.1 kJ/mol. The black curves represent the calculated total angular distribution when the weighted best-fit CM functions of Figure 5 are used for the O(^3^P) and O(^1^D) contributions to the OC_3_N product (top panel) and to the OC_3_N and HCCN products (bottom panel). The relative contributions from the O(^3^P) and O(^1^D) reactions to *m*/*z* = 66 (top panel) and *m*/*z* = 38 (bottom panel) are indicated (color coding as in Figure 1).

**Figure 3:**
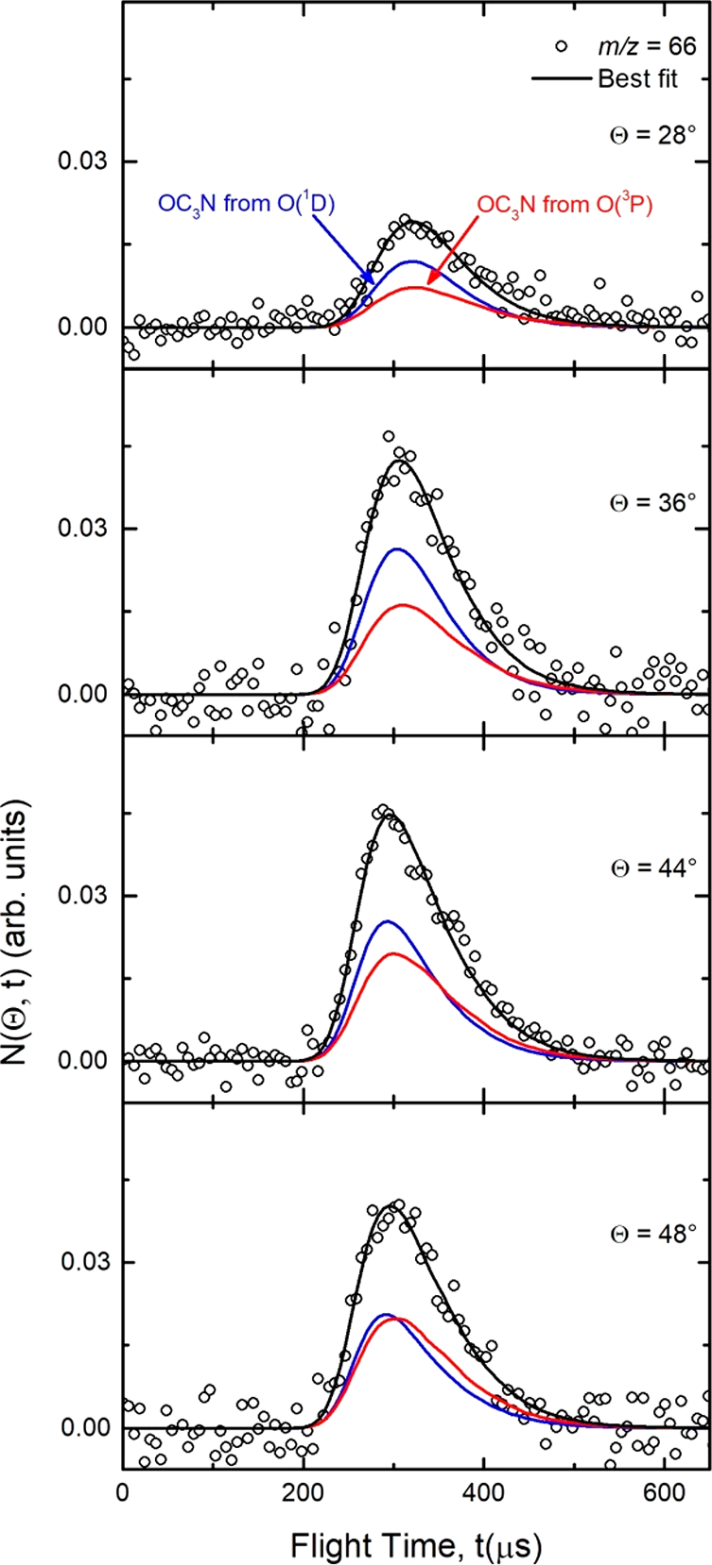
Product LAB time-of-flight distributions measured at *m*/*z* = 66 at four indicated LAB angles for the reactions O(^3^P, ^1^D) + HC_3_N at *E*_c_ = 31.1 kJ/mol. Open circles: experimental data. Black curves: calculated total TOF distributions when using the weighted best-fit CM functions of Figure 5 for the O(^3^P) and O(^1^D) contributions to OC_3_N formation. The distinct contributions from the O(^3^P) and O(^1^D) reactions to the calculated total TOF distributions at each LAB angle are also indicated (line and color notations as in Figures 1 and 2).

The *m*/*z* = 66 LAB angular distribution is reported in Figure 2 (top panel). The filled circles indicate the product intensity averaged over five different scans (with a counting time of 50 s at each angle), while the error bars represent the ±1 standard deviation. The signals at *m*/*z* = 66 corresponds to the parent ion of the heavy coproduct (OC_3_N) of the H-displacement channels (4a and 4b). As can be seen in Figure 2 (top panel), the angular distribution is bell-shaped and narrow; it ranges from 18° to 68° and is peaked around the CM angle (Θ_CM_ = 44.1°). Product TOF distributions at *m*/*z* = 66 were recorded at four different angles (Θ = 28°, 36°, 44°, and 48°) and are shown in Figure 3 (counting time of ca. 2 h at each angle). The single peak structure (peak position around 300 μs) is what is expected from the heavy coproduct of the possible H-displacement channel 4a and possibly also 4b (from O(^1^D)). The contribution of OC_3_N from channels 4a and 4b is also visible, through its daughter ion C_2_N^+^, in the LAB distributions recorded at *m*/*z* = 38 (see Figures 2 (bottom panel) and 4) and will be examined in section 4.3. To fit the data at *m*/*z* = 66 (Figures 2 (top panel) and 3), it was necessary to use the two sets of CM functions shown in Figure 5 and that can be associated with the H-displacement channels 4a, leading to OC_3_N from O(^3^P) on the triplet PES, and 4b, leading to OC_3_N from O(^1^D) on the singlet PES. In principle, there could also be some contribution of OC_3_N from O(^3^P) via ISC, but this is very hard to evaluate.

**Figure 4:**
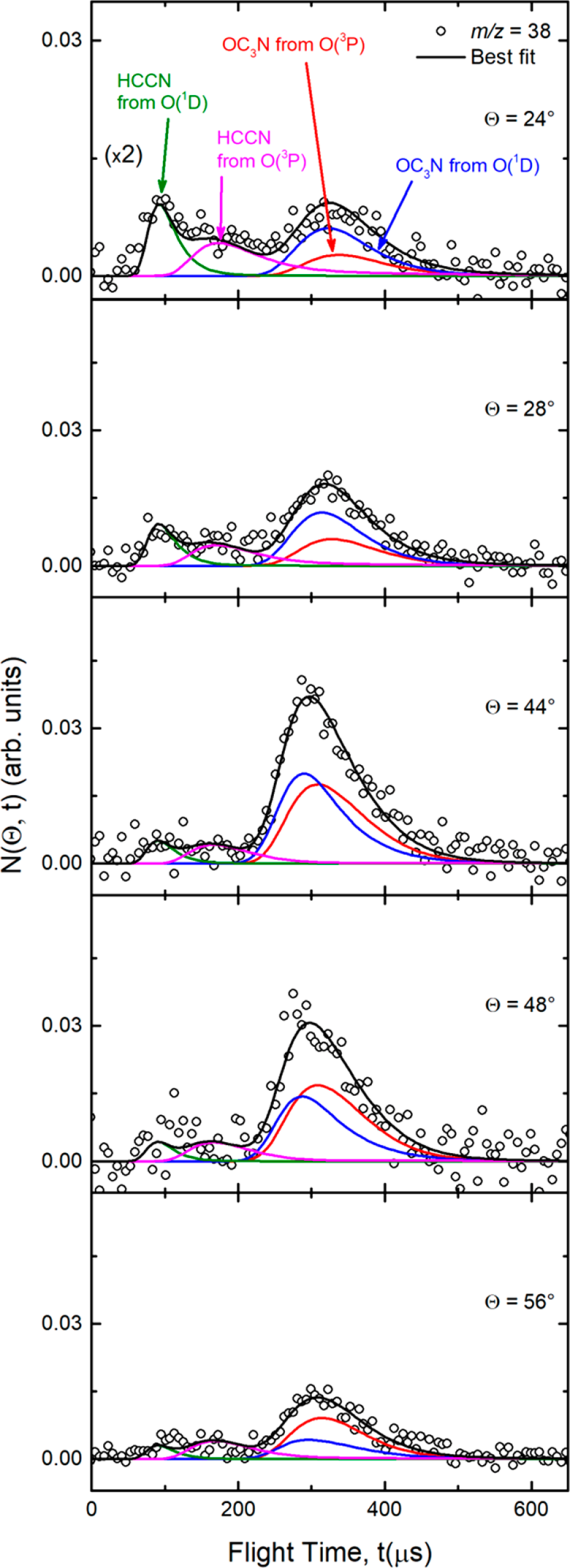
Product time-of-flight distributions measured at *m*/*z* = 38 at five indicated LAB angles for the reactions O(^3^P, ^1^D) + HC_3_N at *E*_c_ = 31.1 kJ/mol. Open circles: experimental data. Black curves: calculated total TOF distributions when using the weighted best-fit CM functions of Figure 5 for the O(^3^P) and O(^1^D) contributions to the OC_3_N and HCCN products. The distinct contributions from the O(^3^P) and O(^1^D) reactions to the calculated global TOF distributions at each LAB angle are also indicated (line and color notations as in Figures 1, 2, and 3). The TOF at Θ = 24° is amplified by a factor of 2.

**Figure 5:**
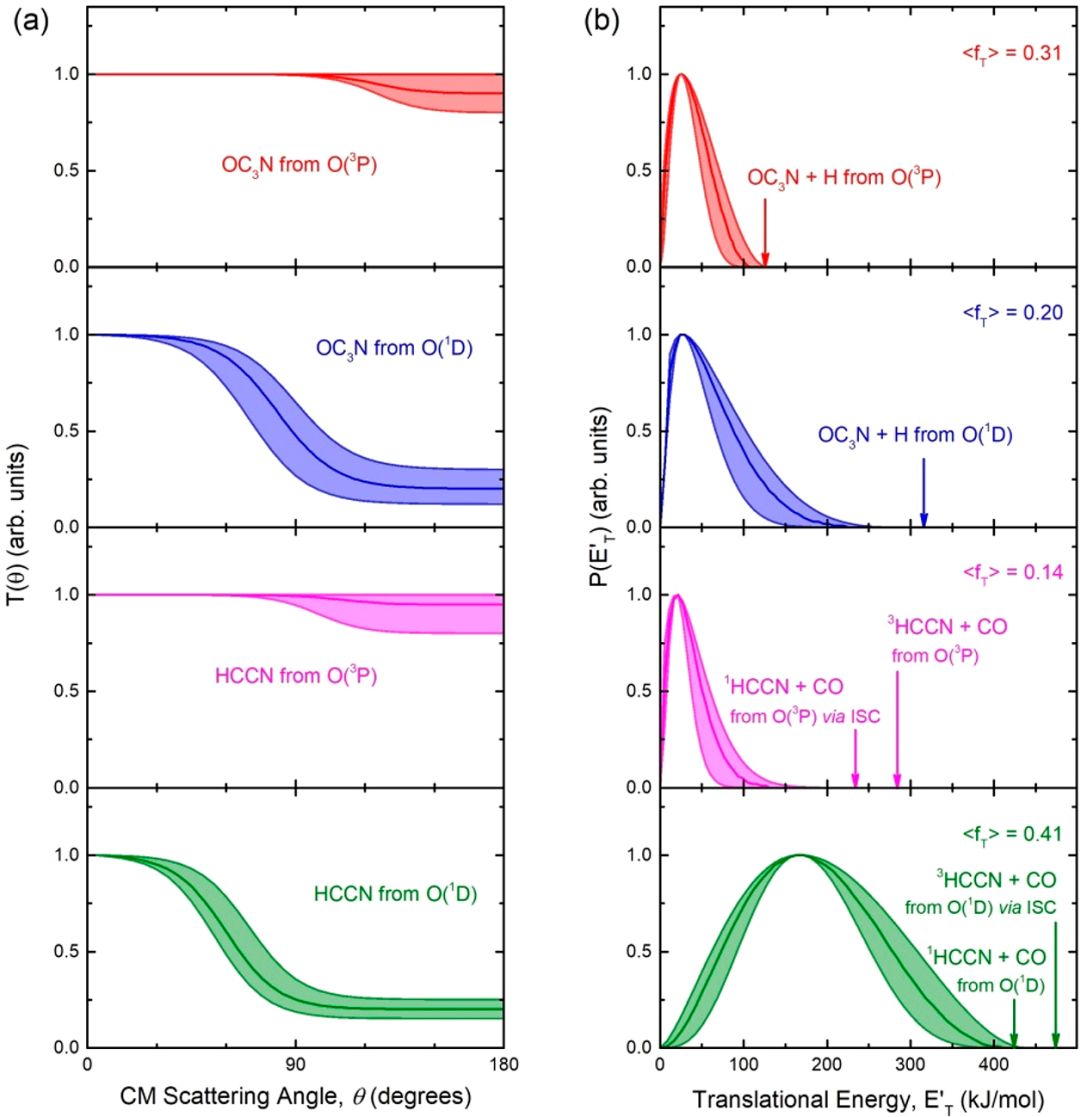
Best-fit center-of-mass angular, *T*(θ) (left panels), and translational energy, *P*(*E*_*T*_^′^) (right panels), distributions for all (four) contributions used to reach the best-fit of the experimental data shown in Figures 2–4 for the O(^3^P, ^1^D) + HC_3_N reactions at *E*_c_ = 31.1 kJ/mol. Line and color notations are as in Figures 1–4. The shaded areas represent the error bars determined for the best-fit CM functions. The solid arrows in the right-hand-side (rhs) panels indicate the total available energy (*E*_TOT_ = Δ*H*_0_^°^ – *E*_c_) for each specified product channel from the O(^3^P) and O(^1^D) reactions. The average fraction of the total available energy, for each channel, released as product translational energy, ⟨*f*_T_⟩, is also indicated.

The solid curves superimposed on the experimental data in Figures 2 and 3 are the simulated distributions when using the best-fit CM angular, *T*(θ), and translational energy, *P*(*E*_*T*_^′^), distributions displayed in Figure 5 for the reaction channels 4a and 4b. As can be seen, while the *T*(θ) of the O(^3^P) reaction is nearly backward–forward symmetric (with only a very slight forward bias), reflecting a *long-lived complex* formation mechanism,^94−96^ that of the O(^1^D) reaction is strongly forward peaked, reflecting an *osculating complex* mechanism,^94−96^ expectedly due to a much shorter lifetime of the decomposing singlet intermediate reached directly from O(^1^D). Indeed, for distinguishing the relative contribution of O(^3^P) and O(^1^D) to the H-forming channels, we have exploited our previous experience on the dynamics of O(^3^P, ^1^D) reactions with several unsaturated hydrocarbons, whereby, because of the much longer lifetime of the triplet intermediate from O(^3^P) with respect to the singlet intermediate from O(^1^D) (see, for the present system, lifetime estimates in sections 6.1 and 6.2, respectively) leading to the same H product channel, the *T*(θ) of the O(^3^P) reaction is symmetric (reflecting a long-lived complex intermediate) while that of the O(^1^D) reaction is forward biased (reflecting an osculating complex intermediate). This approach lead us to the use of the best-fit functions reported in Figure 5 for the H channels from O(^3^P) and O(^1^D) with a comparable relative branching fraction (see Table 1).

**Table 1:** Relative Contributions to the Total Recorded Reactive Signal of the Indicated Unique Sets of Products and Reactants (Second-to-Last Column) and Product Branching Fractions (BFs) for the Distinct O(^3^P) and O(^1^D) Reactions (See Text) (*E*_c_ = 31.1 kJ/mol) (Last Column)^a^

reactants	products	contribution to the total recorded reactive signal	experimental BF
O(^3^P) + HCCCN	^3^HCCN + CO and/or ^1^HCCN + CO (channels 1a and 2a)	0.34 ± 0.10	0.90 ± 0.05
	OC_3_N + H (channel 4a)	0.04 ± 0.02	0.10 ± 0.05
O(^1^D) + HCCCN	^1^HCCN + CO and/or ^3^HCCN + CO (channels 1b and 2b)	0.58 ± 0.17	0.94 ± 0.03
	OC_3_N + H (channel 4b)	0.04 ± 0.02	0.06 ± 0.03

a The experimental uncertainties, ranging from 30% to 50%, are also indicated.

The *P*(*E*_*T*_^′^) distribution for the O(^3^P) reaction (Figure 5, top panel on the rhs) extends up to the limit of energy conservation for channel 4a and is characterized by a large fraction (⟨*f*_T_⟩ = 0.31) of the total available energy released in product translational energy; this indicates the presence of a substantial exit potential barrier in the PES on the way to products (channel 4a). In contrast, the *P*(*E*_*T*_^′^) of the O(^1^D) reaction reflects a substantially smaller average fraction of energy in product translation (⟨*f*_T_⟩ = 0.20), suggesting the absence of a sizable exit barrier in the singlet PES for channel 4b. We recall that ⟨*f*_T_⟩ = ⟨*E*_*T*_^′^⟩/*E*_TOT_, where the average product translational energy, ⟨*E*_*T*_^′^⟩, is defined as ⟨*E*_*T*_^′^⟩ = , and *E*_TOT_ = *E*_c_ – Δ*H*_0_^°^.

### 4.3 The *m*/*z* = 38 Data: The ^1^HCCN (Cyanomethylene) + CO Spin-Forbidden Channel from the O(^3^P) Reaction

The product angular distribution at *m*/*z* = 38 is shown in Figure 2 (bottom panel). The filled circles indicate the intensity averaged over five different scans (with a counting time of 100 s at each angle), while the error bars represent the ±1 standard deviation. The LAB angular distribution is characterized by the same prominent peak centered around Θ_CM_ of the *m*/*z* = 66 distribution, but it is quite clear that it is not confined between 18° and 68° having significant intensity in the two wings. Product TOF distributions at *m*/*z* = 38 were recorded at five different angles (Θ = 24°, 28°, 44°, 48°, and 56°) and are shown in Figure 4 (counting times were from 2 to 4 h at each angle, depending on the signal intensity). In the TOF spectra, it is even more evident that, in addition to the pronounced peak centered at about 300 μs due to the fragmentation of OC_3_N product in the ionizer, there are two distinct fast peaks (very well visible in the forward direction at Θ = 24° and 28°). The fastest peak is located at about 90 μs and the other at about 180 μs. The wings of the *m*/*z* = 38 angular distribution and the two fast peaks in the *m*/*z* = 38 TOF spectra could only be fitted by invoking two additional reactive contributions that, on the basis of energy and linear momentum conservation, can be unambiguously attributed to the HCCN products from the 1a, 2a, 1b, and 2b channels.

Analyzing further the *m*/*z* = 38 TOF spectra, it should be noted that the fingerprints of the HCCN + CO channels are clearer at Θ = 24° than near the CM angle (Θ = 44°), because at Θ_CM_ the relative contributions of the heavy coproducts of the H-displacement channels (4a and 4b) have the maximum relative intensity with respect to HCCN and are strongly amplified in the LAB system for kinematic reasons.^58^ The fact that the LAB angular distribution of HCCN is much wider and the TOF peaks attributed to HCCN are much faster than those of OC_3_N is then due to a combination of the different kinematics and larger exothermicity.

### 4.4 Best-Fit *T*(θ) and *P*(*E*_*T*_^′^) Functions and Reaction Mechanism

Quantitative information on the reaction dynamics is obtained by moving from the LAB frame to the CM frame and analyzing the product *T*(θ) and *P*(*E*_*T*_^′^) distributions into which the total CM product flux can be factorized (see Section 2). The black curves superimposed onto the experimental results in Figures 2–4 are the total calculated LAB angular and TOF distributions (at the indicated *m*/*z*) when using the best-fit CM functions *T*(θ) and *P*(*E*_*T*_^′^) depicted in Figure 5 for each channel. In Figures 2–4, the partial contributions of the various contributing channels at the indicated *m*/*z* value are also indicated with the name of the product as well as with color coding.

Regarding the product translational energy distributions, the best-fit *P*(*E*_*T*_^′^) for channel 4a (see Figure 5) exhibits a peak around 25 kJ/mol, an indication that this channel has an exit potential energy barrier. In addition, it extends up to the total available energy (*E*_TOT_ = *E*_c_ – Δ*H*_0_^°^ = 126 kJ/mol), while the average product translational energy, ⟨*E*_*T*_^′^⟩, is 39 kJ/mol, corresponding to an average fraction, ⟨*f*_T_⟩, of the total available energy released in translation of 0.31. This means that about 70% of the total energy is released as internal (ro-vibrational) energy of the newly formed products.

In contrast, the *P*(*E*_*T*_^′^) of the HCCN + CO channel has a cutoff at about 130 kJ/mol, that is a value much lower than the total energy available for reaction channels 1a and 2a and ⟨*E*_*T*_^′^⟩ is only 35 kJ/mol corresponding to ⟨*f*_T_⟩ = 0.14. Therefore, about 86% of the total available energy is channeled into internal (ro-vibrational and possibly electronic) excitation of the HCCN and CO products. The peaking of the *P*(*E*_*T*_^′^) at about 20 kJ/mol for the HCCN + CO channel from O(^3^P) might indicate the presence of a very low exit potential barrier. Experimentally, we cannot establish whether HCCN is formed in its ground electronic state, ^3^HCCN, or in its first electronically excited state, ^1^HCCN, because the *P*(*E*_*T*_^′^) extension is well within the total energy for both channels (see Figure 5, third panel from the top on the rhs). However, the small value of ⟨*f*_T_⟩ is compatible with the formation of excited ^1^HCCN (the ^1^HCCN–^3^HCCN energy separation is about 50 kJ/mol).

Regarding the O(^1^D) + HC_3_N reaction, the *T*(θ) function for the H-displacement channel (4b) is strongly forward peaked with an intensity ratio, *T*(Θ = 180°)/*T*(Θ = 0°), of only 0.25 (see Figure 5), indicating an osculating complex mechanism,^96−98^ whereby the lifetime of the singlet intermediate accessed adiabatically in the O(^1^D) reaction is shorter than its rotational period. The corresponding best-fit *P*(*E*_*T*_^′^) peaks at about 28 kJ/mol and extend up to about 220 kJ/mol. The average product translational energy is about 62 kJ/mol, which reflects an average fraction of total available energy in product translation, ⟨*f*_T_⟩, of only 0.20. The small fraction of energy released in product translational energy is compatible with the absence of a sizable exit barrier on the singlet PES.

Similarly to the *T*(θ) of the OC_3_N + H channel (4b), also the *T*(θ) of the ^1^HCCN + CO channel (2b) is strongly forward peaked (Figure 5), indicating an osculating complex mechanism. Interestingly, the *P*(*E*_*T*_^′^) distribution of the HCCN-forming channel peaks very far away from zero, at 167 kJ/mol, and dies off at about the total available energy for the ^1^HCCN + CO channel of about 425 kJ/mol. In this case, the average fraction of total available energy released in product translational energy is quite large (⟨*f*_T_⟩ = 0.41), leaving a fraction of about 0.6 for internal excitation of the HCCN + CO products. This large fraction (0.41) of total available energy released in translation could be the result of the presence of an exit barrier or could be due to a nonstatistical redistribution of the total available energy (see the Discussion). We note that a similar trend, that is, the *P*(*E*_*T*_^′^) of the CO channel formed from O(^1^D) peaking at substantially higher energy than the *P*(*E*_*T*_^′^) of the same channel formed from O(^3^P), and correspondingly exhibiting also a substantially larger fraction, ⟨*f*_T_⟩, of the total available energy released in translation, has also been found in the study of the O(^3^P, ^1^D) + benzene reaction at a similar collision energy, for which ⟨*f*_T_⟩ is 0.08 for the C_5_H_6_ + CO channel from O(^3^P) and 0.27 (about 3 times larger) for the same channel from O(^1^D) (see ref (18)).

### 4.5 Product Branching Fractions (BFs)

After the derivation of the best-fit CM *T*(θ) and *P*(*E*_*T*_^′^) functions for the various product channels (Figure 5), the branching fraction of each primary product channel was estimated by using the procedure introduced by Schmoltner et al.^97^ and recently employed by us in the study of a variety of multichannel reactions of O(^3^P) with unsaturated hydrocarbons.^5−19^ In particular, once the origin of the various ion signals from our experimental data is sorted out, the reactive signal associated with a unique set of products and reactants can be derived from the relative apparent cross section (the *w*_*i*_ parameters in the equation of section 2, obtained from the best-fit analysis of the LAB data), the estimated ionization cross section, and the measured total ion yield for a specific product, taking into account the quadrupole mass filter transmission. The experimentally derived relative yields (which have uncertainties ranging from ±30% to ±50% depending on the channel) of the product channels from both the O(^3^P) and O(^1^D) reactions at *E*_c_ = 31.1 kJ/mol are reported in Table 1 (second-to-last column of Table 1). The ionization cross sections at their maximum (70 eV) for the OC_3_N and HCCN products have been evaluated using the procedure of Fitch and Sauter,^98^ which is based on the additivity of atomic ionization cross sections. The ratios of the ionization cross sections of the two different species (OC_3_N and HCCN) are assumed to be the same at 70 and 28 eV (this is an approximate procedure, but it is acceptable with the associated uncertainties that are within those of the overall procedure which can be as large as 50% as quoted above).

As can be seen in Table 1 that the overall contribution of the O(^1^D) reaction channels to the observed reactive signal is 1.63 times (=0.62/0.38) that of the O(^3^P) reaction channels. This fraction depends on the relative concentration of O(^3^P) and O(^1^D) in the atomic beam and on the relative integral cross sections of the reactions involving one of the two atomic states of oxygen with HC_3_N (which are not known). This aspect will be discussed in section 6.3.

From the relative contributions of Table 1, we have obtained the products BFs from the distinct O(^3^P) + HC_3_N and O(^1^D) + HC_3_N reactions (last column of Table 1) by simply normalizing to unity, separately, the sum of the relative fractions of the O(^3^P) channels and of the O(^1^D) channels. As can be seen, the dominant product channel of both the O(^3^P) and O(^1^D) reactions is that leading to HCCN + CO (channels 1a and 2a and channels 1b and 2b, respectively) which exhibits a BF ≥ 90%, while the channels 4a and 4b are minor (≤10%) for both reactions.

## 5 Theoretical Results

### 5.1 Description of the Triplet and Singlet PESs

**Figure 6:**
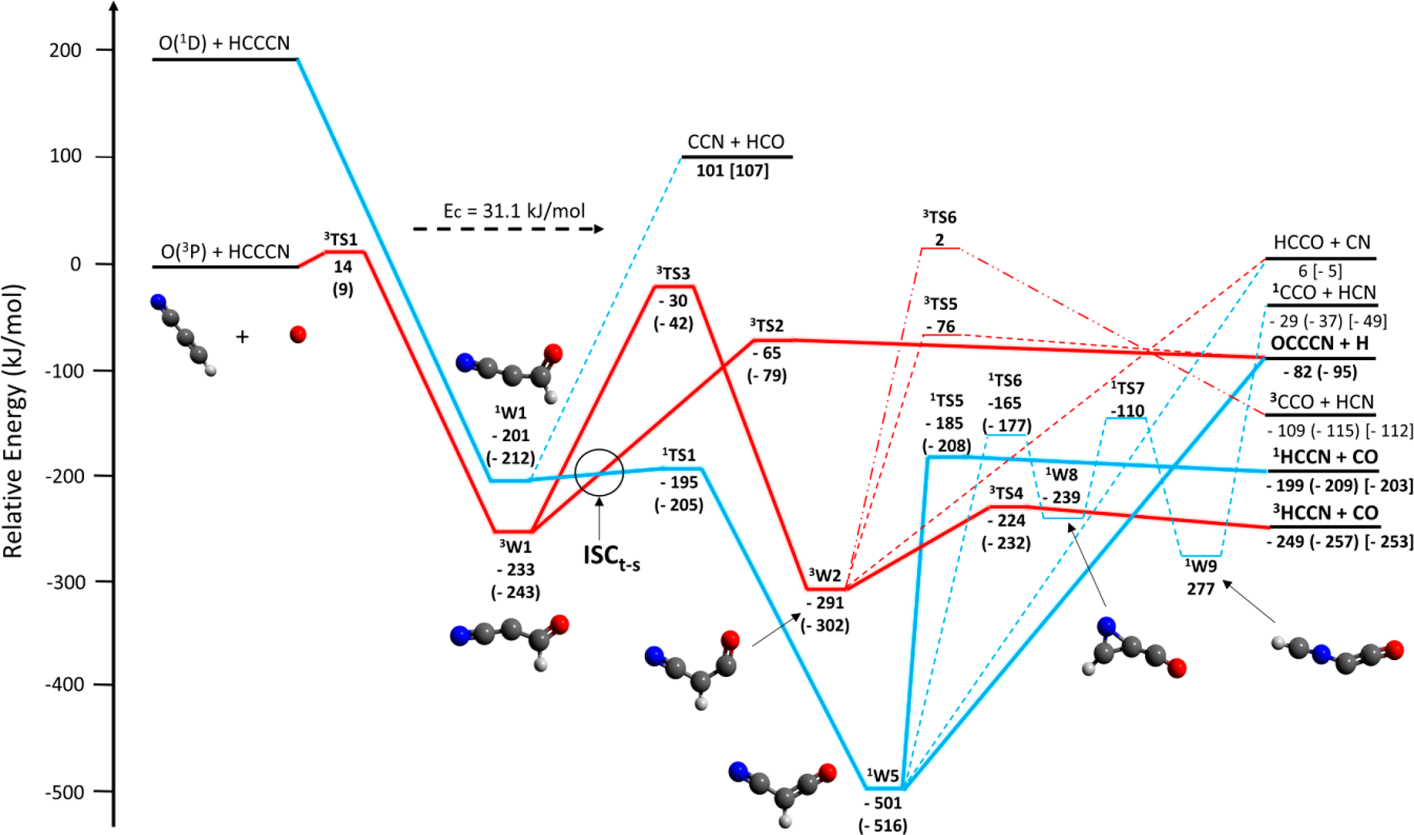
Schematic triplet (red lines) and singlet (blue lines) potential energy surfaces for the O(^3^P, ^1^D) + HC_3_N reactions calculated at the CCSD(T)/aug-cc-pVTZ//B3LYP/aug-cc-pVTZ level of theory. In parentheses, the CBS + core–valence correlation energies are reported. Energies are expressed in kJ/mol with respect to the energy level of the O(^3^P) + HC_3_N reactants. For the sake of simplicity, a few endothermic pathways have been neglected in both PESs. Notably, only the O(^3^P)-addition to the C1 carbon (the one attached to the H) has been illustrated, since the other addition pathways as well as the H-abstraction pathway show substantial entrance energy barriers, inaccessible at the collision energy of 31.1 kJ/mol. The reaction pathways explored by the reactive flux are highlighted in bold red and blue solid lines (dashed lines indicate energetically unfavored pathways). The dashed-double dot pathway from ^3^W_2_ to ^3^CCO + HCN products is a simplified one (see the triplet PES in Figure S1 of the Supporting Information for the details of all isomeric intermediates from ^3^TS6 to products). Predicted products are highlighted in bold black as well. The intersystem crossing (ISC) region where the surface-hopping from the triplet to the singlet PES (ISC_t–s_) in the entrance channel is more likely to occur, is indicated with a circle.

A simplified scheme of the triplet (red lines) and singlet (blue lines) PESs for the bimolecular reactions between O(^3^P, ^1^D) and HC_3_N is depicted in Figure 6. More detailed triplet and singlet PESs are reported in the Supporting Information. All reported energies have been calculated at the CCSD(T)/aug-cc-pVTZ//B3LYP/aug-cc-pVTZ level with the zero-point energy correction computed at the B3LYP/aug-cc-pVTZ level (section 3). The energies of the main relevant reactive pathways computed at the CBS level with inclusion of core–valence correlation are also reported in parentheses.

Since the CCSD(T)/aug-cc-pVTZ//B3LYP/aug-cc-pVTZ calculated barrier for H abstraction in O(^3^P) + HC_3_N is found to be lying 154 kJ/mol above the reactant energy level (see Figure S1 in the Supporting Information), which is much higher than the entrance barrier of 14 kJ/mol calculated (at the same level of theory) for the O-addition channel to the C1 carbon (that bound to the H atom), the PES of Figure 6 does not include the H abstraction pathway. Furthermore, the channels originating from the O-addition to the C2 and C3 carbons of cyanoacetylene, as well as to nitrogen, are also not depicted in Figure 6 because they are not accessible at the collision energy of this experiment (see Figure S1 in the Supporting Information). For the sake of simplicity, also all of the pathways that resulted in being highly endothermic are not reported in Figure 6. For further insights, one can refer to the Supporting Information where a complete representation of the distinct triplet and singlet PESs, including the endothermic channels, is shown.

Let us examine the triplet/singlet PESs in Figure 6. The most favorable initial step (i.e., having the lowest entrance barrier—see Figure S1 of the Supporting Information) is the O(^3^P) addition to the carbon atom (C1) bonded to hydrogen. As already mentioned, a barrier (^3^TS1) of 14 kJ/mol has been calculated for this addition step. This value is in good agreement with the best value previously reported by Xie et al.^49^ who computed this barrier using several methods, that is, G3//B3LYP/6-31G(d), G3//BH&HLYP/6-31G(d,p), G3//MP2(full)/6-31G(d), G3//BH&HLYP/6-311++G(d,p), CASPT2(14,12)/cc-pVDZ//CASSCF(14,12)/cc-pVDZ, G3//QCISD/6-31G(d), and G3//QCISD/6-31+G(d,p), and obtained values ranging from 14 to 21 kJ/mol. At the highest level of calculations employed, CASPT2(14,12)/cc-pVDZ//CASSCF(14,12)/cc-pVDZ, their value for ^3^TS1 is 15 kJ/mol. However, this value seems to be somewhat high if one considers the entrance barriers for similar systems, like for instance O(^3^P) + propyne, where an entrance barrier of only 7 kJ/mol was computed at the CASPT2 level.^11,12^ For this reason, we computed this energy barrier at the CBS level with inclusion of core–valence correlation and obtained a smaller value of 9 kJ/mol. The energy of the other relevant stationary points along the minimum energy paths for the singlet and triplet PESs have therefore been calculated at the same level. In the following discussion, the CBS + core–valence correlation energies for the main reaction channels are reported in parentheses (also indicated in Figure 6).

After the O addition to C1, a bound intermediate (^3^W1), associated with an energy well of −233 (−243) kJ/mol below the energy of the reactants (Figure 6), is formed. ^3^W1 can either dissociate directly to the products OCCCN + H by overcoming a transition state, ^3^TS2, located at −65 (−79) kJ/mol or undergo a 1,2 H-shift by overcoming a barrier (^3^TS3, located at −30 (−42) kJ/mol with respect to the reactants) with the formation of a second bound intermediate, ^3^W2. Located at 291 (302) kJ/mol below the reactants, ^3^W2 can in turn dissociate into HCCO + CN, OCCCN + H, or ^3^HCCN + CO or isomerize to ^3^W7 (see Figure S1 in the Supporting Information). The C–C bond breaking channel with the formation of HCCO + CN (channel 6a) is endothermic by 6 kJ/mol (or slightly exothermic by −5 kJ/mol). The breaking of the H–C bond in the ^3^W2 intermediate occurs via a transition state, ^3^TS5, located at −76 kJ/mol with respect to the reactants, and leads to the OC_3_N + H products. This channel is exothermic by 82 (95) kJ/mol. The other C–C bond breaking channel leading to ^3^HCCN + CO is exothermic by 249 (257) kJ/mol and requires overcoming a small exit barrier (^3^TS4) located at +25 kJ/mol above the products. Finally, the H-shift to the nitrilic carbon leads to ^3^W7 located at −184 kJ/mol below the reactants followed by a number of isomerizations ending up with ^3^CCO + HCN production (cf. the triplet PES in the Supporting Information), by C–C bond cleavage. This channel is exothermic by 109 (115) kJ/mol and was not reported by Xie et al.^49^

We note that most of the energetics of the stationary points in Figure 6 are higher in energy if compared to those calculated at the G3//B3LYP/6-31G(d) and G3//MP2(full)/6-31G(d) levels of theory by Xie et al.^49^ The differences, however, are within 10 kJ/mol; that is, they fall within the accuracy of these methods.

The singlet PES for the reaction of HC_3_N with atomic oxygen in its first excited state, O(^1^D), is also shown in Figure 6 (blue lines). The reported energies have been calculated at the same level of theory as the triplet PES. The O(^1^D) + HC_3_N reactant energy was determined by adding the experimental value of 190 kJ/mol for the energy difference between the O(^3^P) ground state and the O(^1^D) excited state,^99^ in contrast to Xie et al.’s study^49^ where the electronic excitation was calculated. Their value was higher by 8 kJ/mol with respect to the accepted experimental value. Differently from the reactions of O(^3^P), the O(^1^D) addition reactions are usually barrierless and this case is not an exception.^18,19^

Four different types of attacks by O(^1^D) on HC_3_N are found for the singlet PES, leading to the barrierless formation of the singlet intermediates ^1^W1, ^1^W2, ^1^W3, and ^1^W4, shown in Figure S1 of the Supporting Information. The most relevant singlet pathway under our experimental conditions is that leading to ^1^W1, as portrayed in Figure 6. Located at −201 (−212) kJ/mol, ^1^W1 can undergo an isomerization reaction to ^1^W5. A 1,2 H-shift can occur by overcoming a small barrier (^1^TS1) of 5 (7) kJ/mol. The resulting singlet intermediate COCHCN (cyanoketene), ^1^W5, is the most stable structure in the PES, lying at −501 (−516) kJ/mol below the O(^3^P) + HC_3_N asymptote. From ^1^W5, the system can evolve by following different pathways. The barrierless cleavage of the C–H bond or of the single C–C bond leads to OCCCN + H and to HCCO + CN, respectively. Otherwise, the breaking of the C–C double bond leads to ^1^HCCN + CO (−199 (−209) kJ/mol). In this case, the system must overcome a barrier (^1^TS5) of 316 (308) kJ/mol (from ^1^W5), which is located at 14 (1) kJ/mol above the products. Finally, a H-shift may occur to the nitrilic carbon by overcoming a barrier of 336 (339) kJ/mol (^1^TS6), leading to ^1^W8 located at −239 (−254) kJ/mol with respect to the reactants, which may isomerize to ^1^W9 via ^1^TS7, that in turn undergoes C–N bond cleavages resulting in ^1^CCO + HCN formation. This channel is exothermic by −29 (−37) kJ/mol and was not reported by Xie et al.^49^

### 5.2 RRKM/Master Equation Adiabatic Simulations of Branching Fractions

The statistically predicted BFs on the adiabatic triplet and singlet PESs for the reactions O(^3^P) + HC_3_N and O(^1^D) + HC_3_N, respectively, are reported in Table 2. If we assume that the reactive flux of the O(^3^P) + HC_3_N reaction occurs adiabatically on the triplet PES, the H-displacement channel (4a) results in being by far the most abundant one with a calculated branching fraction of 0.98 at *E*_c_ = 31.1 kJ/mol, while the C–C bond breaking channel leading to ^3^HCCN + CO (channel 1a) is minor (BF = 0.02). By comparing the two adiabatic predictions with the experimental values, we can conclude that the extent of ISC is ca. 90% under the conditions of our CMB experiment.

**Table 2:** Theoretical Branching Fractions Calculated for the O(^3^P) + HC_3_N Reaction at *E*_c_ = 31.1 kJ/mol Occurring Adiabatically on the Triplet PES (Second Column) and for the O(^3^P) Reaction Assuming Complete ISC to the Underlying Singlet PES at the Collision Energy of the Present CMB Experiment^a^

products	O(^3^P) reaction on the triplet PES (adiabatic)	O(^3^P) reaction proceeding only via ISC	assuming an ISC extent of 90%	experimental BF
HCCN + CO	0.02 (^3^HCCN)	0.99 (^1^HCCN)	0.89	0.90 ± 0.05
OC_3_N + H	0.98	0.01	0.11	0.10 ± 0.05

a The best comparison with the experimental BF values (last column) is obtained when assuming that 90% of the reaction proceeds via ISC.

For the O(^1^D) + HC_3_N reaction, adiabatic RRKM calculations on the singlet PES predict the CO-forming channel (2b) (^1^HCCN + CO) to be the dominant active channel, with a theoretical BF of 0.97, whereas the BF of the H-displacement channel (OC_3_N + H) is 0.03. The RRKM results are in excellent agreement with the experimental determination (see the last column of Table 1).

## 6 Discussion

### 6.1 Dynamics of the O(^3^P) + HC_3_N Reaction

The best-fit angular distributions in the CM system for the OC_3_N + H channel 4a and for the HCCN + CO channel 1a and/or 2a from the O(^3^P) reaction with HC_3_N are, within the error bounds, both nearly backward–forward symmetric (nearly isotropic, with a slight bias in the forward direction) (see Figure 5). This is typical for a reaction proceeding via a *long-lived* complex mechanism. In the light of the electronic structure calculations, this observation is consistent with the formation of a bound intermediate (^3^W1) after the O(^3^P) addition to the C1 atom. Considering the nearly complete backward–forward symmetry of the *T*(θ), the lifetime of ^3^W1 should be ≥5–6 its rotational period according to the *osculating* model of chemical reactions.^94−96^ This is in line with the strong stability (−233 kJ/mol) of the ^3^W1 intermediate (theoretically estimated lifetime of ∼450 ps at *E*_c_ = 31.1 kJ/mol) that can (i) unimolecularly decay adiabatically (on the triplet PES) to OCCCN + H via ^3^TS2 and/or to ^3^HCCN + CO through isomerization via ^3^TS3 to ^3^W2 and then dissociation to ^3^HCCN + CO via ^3^TS4, or (ii) undergo ISC to ^1^W1 at the seam of intersection between ^3^W1 and ^1^W1 (indicated qualitatively with a circle in Figure 6). The singlet ^1^W1 intermediate can then quickly isomerize to the very stable (−516 kJ/mol) ^1^W5 (cyanoketene) that can unimolecularly decay (because of its high internal energy content at *E*_c_ = 31.1. kJ/mol) to ^1^HCCN + CO via a relatively *loose*^1^TS5 transition state, and/or to OCCCN + H barrierlessly. The energetics of the competing product channels are such that, experimentally, it is not possible to distinguish whether ground-state ^3^HCCN or electronically excited ^1^HCCN is formed, because the cutoff of the *P*(*E*_*T*_^′^) distribution for the HCCN + CO channel from the O(^3^P) reaction is less than one-half of the total available energy for the ^1^HCCN + CO channel and even more so of that for the ^3^HCCN + CO channel (which is about 50 kJ/mol more exothermic than ^1^HCCN + CO, due to the higher stability of ground-state ^3^HCCN with respect to ^1^HCCN) (see Figure 5, third panel from the top on the rhs).

According to our RRKM/ME calculations of product BFs, if the O(^3^P) + HC_3_N reaction evolves adiabatically on the triplet PES at the *E*_c_ of the experiment, the predicted BFs are 0.98 for the H-displacement channel and 0.02 for the CO formation channel (Table 2). Therefore, since the experimental BFs clearly indicate that HCCN + CO is the dominant channel, ISC to the singlet PES to a significant extent must be called into play. Since the experimental ratio (HCCN + CO)/(OC_3_N + H) is 0.90/0.10 (=9), rather than the adiabatically predicted 0.02/0.98 (≈0.02) (see Table 2), that is, a factor of 450 larger, we conclude that nearly all HCCN formed from the O(^3^P) reaction is actually the spin-forbidden excited cyanomethylene, ^1^HCCN, from channel 2a reached via ISC, rather than ground-state ^3^HCCN from the adiabatic channel 1a. The main reaction channel is the C–C bond breaking channel forming CO and HCCN, and this means that the three-carbon chain of cyanoacetylene is not maintained when attacked by O(^3^P) (by an extent of 90%), and this is due to efficient triplet to singlet ISC in the entrance channel.

It is worth comparing the present conclusions with the suggestion put forth in the theoretical work of Xie et al.^49^ These authors concluded that the dominant product channel of the O(^3^P) + HC_3_N reaction is the adiabatic ^3^HCCN + CO channel (1a). Our experimental results and statistical adiabatic estimates of the product distribution on the *ab initio* triplet PES strongly indicate that triplet to singlet ISC in the entrance channel is very efficient in the title reaction, because formation of ^3^HCCN is highly unfavored on the triplet PES (see Table 2) and the dominant product channel is ^1^HCCN + CO from the O(^3^P) reaction, which is also consistent with the shape of the corresponding *P*(*E*_*T*_^′^) distribution, as already discussed. Having observed both product channels (H- and CO-forming channels), we can conclude that the extent of ISC is about 90%, which is similar to what was observed (about 85%) in the related O(^3^P) + HCC–CH_3_ (propyne) reaction (see refs (11 and 12) and section 6.4). What we do not know experimentally is whether additional singlet to triplet ISC is occurring in the exit channel from ^1^W5 to ^3^W2 (see Figure 6) that could lead to production of ^3^HCCN + CO. To answer these questions, a detailed treatment of ISC in both the entrance and exit channels of the O(^3^P) + HC_3_N reaction would be necessary, but this is outside the scope of the present work and is left for future theoretical efforts. In any case, the excited ^1^HCCN product is expected to decay spontaneously to ground-state ^3^HCCN, and ultimately the main product of the O(^3^P) + HC_3_N reaction is ground-state ^3^HCCN (+ CO). This is particularly relevant also in astrophysical environments, where it is ground-state ^3^HCCN that has actually been observed.

It is interesting to also examine the present results on the O(^3^P) + HC_3_N reaction along with previous results on the O(^3^P) reactions with a variety of unsaturated hydrocarbons. From the trend of the extent of ISC in related O(^3^P) + unsaturated hydrocarbon reactions, where ISC of variable extent (ranging from about 20% in O(^3^P) + propene^10^ up to >90% in O(^3^P) + allene^13^), in the vicinity of the minimum of the initial triplet diradical intermediate, has been observed, we expect that the extent of ISC also in the O(^3^P) + HC_3_N reaction will increase with decreasing collision energy (temperature) because the lifetime of the intermediate will also increase at lower *E*_c_ (temperature), thus increasing the probability of ISC.^100^ The opposite will occur at higher *E*_c_ (temperatures), more relevant to combustion environments; in fact, at high combustion temperatures, we expect that the H-displacement channel will increase in importance and that the cyanoketyl product will play a larger role than at low *E*_c_ (temperatures).

### 6.2 Dynamics of the O(^1^D) + HC_3_N Reaction

Regarding the O(^1^D) + HC_3_N reaction, the best-fit angular distributions in the CM system for the OC_3_N + H channel (4b) and for the ^1^HCCN + CO channel (2b) are both strongly forward peaked. The backward to forward intensity ratio of about 0.25 is typical of reactions proceeding via an *osculating* complex mechanism.^94−96^ That is, the intermediate singlet complex ^1^W5, formed following O(^1^D) addition to the C1 atom of the triple C≡C bond of HC≡C—CN forming initially ^1^W1 (−201 (−212) kJ/mol) which quickly isomerizes to ^1^W5 (−501 (−516) kJ/mol), has a complex lifetime, τ, considerably shorter (the calculated value is <5 ps) (because of the extra 190 kJ/mol of internal energy in the complex) than that of ^3^W1 (−233 (−243) kJ/mol) from the O(^3^P) reaction, and in particular shorter than the singlet complex rotational period (estimated to be about 4 ps). Specifically, the very pronounced backward–forward asymmetry of the *T*(θ) of 0.25 corresponds to a ratio τ/τ_rot_ slightly smaller than unity, according to the (approximate) *osculating* model of chemical reactions.^94−96^

It should be noted that the ratio of the CO/H channel yield for the O(^1^D) reaction is experimentally derived to be (0.94 ± 0.03)/(0.06 ± 0.03) and this value, within the error bars, is very similar to the adiabatic calculated ratio of 0.97/0.03 (see Table 3). This appears to indicate that the O(^1^D) reaction proceeds adiabatically on the singlet PES. However, also in this case, we do not know whether there is efficient ISC in the exit channel from the singlet to the triplet PES leading to formation of ^3^HCCN + CO rather than ^1^HCCN + CO. In any case, as for the O(^3^P) reaction, ^1^HCCN would spontaneously decay to the ground state and ultimately the products will be ^3^HCCN + CO also from the O(^1^D) reaction.

**Table 3:** Theoretical Branching Fractions Calculated for the O(^1^D) Reaction Occurring Adiabatically on the Singlet PES, Compared to the Experimental BFs at the Collision Energy of the Present CMB Experiment^a^

products	O(^1^D) reaction on the singlet PES (adiabatic)	experimental BF
HCCN + CO	0.97 (^1^HCCN)	0.94 ± 0.03
OC_3_N + H	0.03	0.06 ± 0.03

a The good agreement, within the error bars, with the experimental BF values indicates that the O(^1^D) reaction proceeds adiabatically on the singlet PES forming ^1^HCCN.

The difference in the shape of the *P*(*E*_*T*_^′^) distribution for the ^1^HCCN + CO channel from O(^1^D) and the ^1^HCCN + CO channel from O(^3^P) is worth some comments (see Figure 5, two bottom panels on the rhs). The much larger fraction of the total available energy channeled in translation in the case of the O(^1^D) reaction (⟨*f*_T_⟩ = 0.41) with respect to that of the O(^3^P) reaction (⟨*f*_T_⟩ = 0.14) should reflect a very high exit barrier. However, the exit barrier is the same experienced in the formation of ^1^HCCN + CO via ISC from the O(^3^P) reaction, and that barrier, ^1^TS5, is actually a very small one (∼1 kJ/mol) with respect to products. This suggests that either a large fraction of the electronic energy of the O(^1^D) atom is channeled into product translational motion or the lifetime of the intermediate is too short to allow for a full energy randomization. Only a detailed theoretical dynamical treatment of entrance and exit channel ISC effects could shed further light on this interesting issue. Unfortunately, this is out of the current capabilities for this complex polyatomic system.

Regarding the less exothermic product channels from the O(^1^D) reaction, because statistical calculations of the BFs from the O(^1^D) reaction indicate for OCCCN + H a BF of only 0.03 with respect to the dominant ^1^HCCN + CO channel (BF = 0.97) (see Table 3), and we have not found, within our experimental sensitivity, a reactive signal at *m*/*z* = 40 (CCO) and 27 (HCN), as well as *m*/*z* = 41 (HCCO) and 26 (CN), we conclude that channels 5b and 6b from O(^1^D) are negligible. We can then reasonably assume that also the less exothermic channels 7b, 8b, and 9b from O(^1^D) are negligible at *E*_c_ = 31.1 kJ/mol.

As a last point, our results on the dynamics of the O(^3^P, ^1^D) + HC_3_N reactions support and help to rationalize the experimental findings by Borget et al.^48^ who investigated on a water ice surface at 7 K the reaction of HC_3_N with atomic oxygen generated from photodissociation of ozone at 255 nm. They observed and characterized the formation of cyanoketene (COCHCN), that is, ^1^W5 in Figure 6. Cyanoketene corresponds to the most stable intermediate on the ground-state singlet PES (see Figure 6) and can be formed by the barrierless (on the water ice surface)^49^ O(^3^P) addition on the triple C≡C bond of HC_3_N, followed by ISC to the ground-state singlet PES and H migration, and then by collisional stabilization. Alternatively, the dominant O(^1^D) species produced by the 255 nm photolysis of O_3_ can directly add to the triple C≡C bond, leading after ready 1–2 H shift to singlet cyanoketene (^1^W5), which is stabilized on the surface.

### 6.3 O(^3^P) versus O(^1^D) Reactivity with HC_3_N

It is useful to take a closer look at the BFs reported in Table 1. If we add all the yields from the O(^3^P) reaction channels (2a, 4a) and those from the O(^1^D) reaction channels (2b, 4b), we find the following ratio: [yield O(^3^P) reactions]/[yield O(^1^D) reactions] = 0.38/0.62 (=0.61); that is, under our experimental conditions, only about 39% of the total reactive signal originates from the O(^3^P) reaction with cyanoacetylene, while the rest (61%) comes from the O(^1^D) reaction. If we assume that the concentration of O(^1^D) in the atomic oxygen beam is about 10% (upper limit) of that of O(^3^P),^65^ this would indicate that at *E*_c_ = 31.1 kJ/mol the total reactive cross section of the reaction of cyanoacetylene with O(^1^D) is about 16 times larger than that with O(^3^P). This is plausible; in fact, given that the barrierless O(^1^D) reaction with cyanoacetylene is expected to be gas-kinetic (*k*_300K_ ≈ 1 × 10^–10^ cc molec^–1^ s^–1^) (with a weak temperature dependence), the present experimental results suggest that the rate constant of O(^3^P) should be about 6 × 10^–12^ cc molec^–1^ s^–1^ at a temperature corresponding approximately to *E*_c_ = 31.1 kJ/mol, which is reasonable considering the calculated entrance barrier of 9 kJ/mol and that the global O(^3^P) rate constant will increase with increasing temperature. With a (calculated) entrance barrier of 9 kJ/mol (at the CCSD(T)/CBS level), the global rate constant at 300 K is then expected to be of the order of 10^–12^ cc molec^–1^ s^–1^. This can be useful information for modelers.

As shown in Table 2 and Table 3, the trends of BFs for the two main competing reaction channels of the O(^3^P) + HC_3_N and O(^1^D) + HC_3_N reactions are found to be significantly different. The fact that in the O(^3^P) reaction there is comparatively more (nearly a factor of 2) H channel than in the O(^1^D) reaction (BF = 0.10 vs 0.06) is due to the fact that for the O(^3^P) reaction the fraction of H channel comes from the adiabatic reaction on the triplet PES, while for the O(^1^D) reaction comes from the competitive dissociation of the singlet intermediate ^1^W5 toward OC_3_N + H and ^1^HCCN + CO (see Figure 6). We remind that in the O(^3^P) reaction the unimolecular dissociation of ^1^W5, reached via ISC, leads to negligible amounts of OC_3_N + H (BF = 0.01) (see Table 2), while the unimolecular decomposition of ^1^W5 at the total energy of the O(^1^D) reaction, at the *E*_c_ of the experiment, is predicted to lead to OCCCN + H with BF = 0.03 and to ^1^HCCN + CO with BF = 0.97 (see Table 3).

Interestingly, the reaction of cyanoacetylene with both O(^3^P) and excited O(^1^D) leads dominantly, via C–C bond cleavage, to ^1^HCCN + CO (BF = 0.90 ± 0.05 and 0.94 ± 0.03, respectively). We expect that the BF of the CO-forming channel for the O(^3^P) reaction, being certainly due to ISC, will increase with decreasing collision energy (and hence with decreasing temperature). This may be relevant for the chemistry of the ISM and of those environments where these reactions are relevant.

### 6.4 Comparison between the O(^3^P) + HCC–CN and O(^3^P) + HCC–CH_3_ Reaction Dynamics

It is interesting and useful to compare the reaction dynamics of O(^3^P) + HC_3_N with that of the related system O^(3^P) + HCC–CH_3_ (propyne), where the CN group is replaced by the CH_3_ group. A recent, detailed, combined CMB and theoretical study from our laboratory of the O(^3^P) + propyne reaction at *E*_c_ = 38.5 kJ/mol found that the addition of O(^3^P) to the terminal carbon of the triple bond is most favored with respect to the addition to the central C.^11,12^ The latter leads mainly to two different competitive reaction pathways: ISC to the singlet PES and decomposition of the triplet intermediate to the strongly exothermic HCCO (ketyl) + CH_3_ channel (Δ*H*_0_^°^ = −112 kJ/mol). Experimentally, the latter radical channel was found to have BF = 0.10 ± 0.05, while the statistical prediction is 0.13.^11,12^ In the case of the O(^3^P) + HC_3_N reaction, the corresponding product channel is HCCO + CN (channel 6a) which is, however, nearly thermoneutral (Δ*H*_0_^°^ = 6 kJ/mol (−5 kJ/mol from the literature enthalpies of formation)); indeed, in the present study, it was not observed to occur experimentally or theoretically. In contrast, O(^3^P) addition to the terminal (C1) carbon of propyne leads to the triplet intermediate *cis*-CH_3_CCHO (and its *trans* isomer).^11,12^ These two isomers can follow mainly four competitive reaction routes: decomposition to CH_3_CCO + H (Δ*H*_0_^°^ = −72.4 kJ/mol) (experimental BF = 0.04 ± 0.02, statistical BF = 0.22), to C_2_H_3_ + HCO (Δ*H*_0_^°^ = −96.7 kJ/mol) (experimental BF = 0.11 ± 0.04, statistical BF = 0.16), to ^3^C_2_H_4_/CH_3_CH + CO (this was not observed experimentally nor predicted statistically), and efficient ISC to the singlet PES forming aldehyde and ketone isomeric intermediates, that lead dominantly to ^1^C_2_H_4_ + CO (experimental BF = 0.74 ± 0.25; statistical BF = 0.35). The corresponding channels in the O(^3^P) + HC_3_N reactions are OC_3_N + H (channel 4a) (BF = 0.10 ± 0.05) (Δ*H*_0_^°^ = −95 kJ/mol) and HCO + C_2_N (channel 7a) (BF = 0) (Δ*H*_0_^°^ = +89 kJ/mol).

The above comparisons indicate that at comparable *E*_c_ in the O(^3^P) + HC_3_N reaction there are only two main product channels, both exothermic: OCCCN + H formation on the triplet PES (BF = 0.10 ± 0.05) and ^1^HCCN + CO formation via ISC to the singlet PES (BF = 0.90 ± 0.05), while the other two corresponding channels of the O(^3^P) + HCCCH_3_ reaction, namely, CH_3_ + HCCO and C_2_H_3_ + HCO, which are CN + HCCO (channel 6a) and C_2_N + HCO (channel 7a), do not occur in the O(^3^P) + HCCCN reaction because they are nearly thermoneutral and substantially endothermic, respectively (these channels are actually negligible also for the O(^1^D) reaction, due to the unfavorable energetics). However, a very noticeable and interesting aspect is that the extent of ISC is comparable in the two reactions, about 90% in O(^3^P) + HCC–CN and about 85% in O(^3^P) + HCC–CH_3_.^11,12^ As discussed in detail in the case of the O(^3^P) + propyne reaction,^11,12^ and examining the C_3_ series of unsaturated hydrocarbons (propene, propyne, and allene),^100^ the above similar behavior is not surprising since it is the long lifetime of the initial triplet intermediate, which in both reactions has a stability of about 220–240 kJ/mol, that determines, at comparable *E*_c_, the similar high probability of ISC from the triplet to the corresponding singlet diradical intermediate.

## 7 Implication for Cosmochemistry

According to the present experimental and theoretical investigation, the O(^3^P) + cyanoacetylene reaction is dominated, at the collision energy of 31.1 kJ/mol, by the spin-forbidden cyanomethylene (^1^HCCN) + CO channel with a BF of 0.90 ± 0.05, while the only other competitive reactive pathway to cyanoketyl (OCCCN) + H is minor (BF = 0.10 ± 0.05). The ratio ^1^HCCN/OCCCN is expected to increase with decreasing *E*_c_ (temperature) because that should facilitate the occurrence of ISC. Given the entrance barrier of 9 kJ/mol (at the CCSD(T)-CBS level), we expect a rate coefficient on the order of the 10^–12^ cm^3^ molec^–1^ s^–1^ range at 300 K. Therefore, the title reaction is expected to be of relevance in warm extraterrestrial environments such as PDR regions, circumstellar envelopes of carbon-rich stars (such as IRC+10216),^101−103^ cometary comae, and, possibly, also in the upper atmosphere of Titan, where some oxygen is present (O^+^ originates from the magnetosphere of Saturn and is quickly transformed into neutral atomic oxygen).^104^

Mechanisms of cyanomethylene formation in space are uncertain.^105^ HCCN has been very recently observed also in TMC-1, a very cold source.^106^ The O(^3^P) + HC_3_N reaction could well represent an additional mechanism of formation of cyanomethylene (HCCN) given the large relative abundance of atomic oxygen and the ubiquitous presence of HC_3_N in the ISM. The title reaction is currently overlooked by modelers. In fact, no information about this reaction is present in astrochemical databases, such as KIDA^107^ and UMIST.^108^

To improve current astrochemical models, we propose to include the O(^3^P) + cyanoacetylene reaction both as a possible destruction pathway of HCCCN and a possible formation route of HCCN, and of also OC_3_N.

Furthermore, atomic oxygen in its first excited state, O(^1^D), has been clearly detected in cometary comae where it is produced by the photodissociation of several parent species (H_2_O and/or CO/CO_2_).^38^ We emphasize here that O(^1^D) is incredibly reactive with closed shell molecules and bimolecular reactions have been recently invoked to explain the formation of detected molecules.^39^ Another reason for being interested in the reaction O(^1^D) + HC_3_N is associated with recent experimental investigations suggesting a possible role of excited ^1^D oxygen atoms in interstellar ice reactions. Reactions of O(^1^D) with organic molecules present in interstellar ices have been invoked to explain the formation of oxygenated organic molecules such as CH_3_OH, H_2_CO, C_2_H_5_OH, CH_3_CHO, CH_2_(O)CH_2_, and CH_2_CO. Similarly, the O(^1^D) + HC_3_N reaction assisted by the water ice surface could lead to the stabilization of cyanoketene, as observed in the experiment by Bogert et al.^48^

In conclusion, the present study on the gas-phase reactions of O(^3^P) and O(^1^D) with HC_3_N can enrich our knowledge of the gas-phase chemistry of nitrile compounds that are key intermediates in the formation of many species with biological potential, such as nucleobases and amino acids, both on Earth and in extraterrestrial environments.^109−113^

## 8 Conclusions

We have reported a combined CMB and theoretical study of the O(^3^P) + cyanoacetylene reaction, a process of considerable relevance in a variety of extraterrestrial environments (including Titan’s atmosphere) as well as in combustion systems. We have determined that the reaction exhibits two main product channels. Specifically, in CMB experiments at a collision energy of 31.1 kJ/mol, it is found that the main reaction channel is that leading to formation of the spin-forbidden ^1^HCCN (cyanomethylene) + CO products (BF = 0.90 ± 0.05), which are formed via efficient ISC from the entrance triplet PES to the underlying singlet PES, while the spin-allowed OCCCN (cyanoketyl) + H product channel, occurring adiabatically on the triplet PES, is minor (BF = 0.10 ± 0.05). The theoretical results have indicated that the dominant reaction mechanism is addition of atomic oxygen to the C1 carbon of the triple C≡C bond of HCC—CN, occurring with an entrance barrier of 9 kJ/mol, and this makes this reaction relevant not only in combustion environments but also in relatively warm regions of the ISM, such as circumstellar envelopes and PDRs, and also the upper atmosphere of Titan, where it could represent an efficient mechanism of formation of cyanomethylene. We recall that the main product of the title reaction, cyanomethylene, has been detected toward IRC+10216 where HC_3_N is particularly abundant and O atoms are present^101−103^ as well as in the upper atmosphere of Titan.

The present study lends us to propose to include the O(^3^P) + cyanoacetylene reaction both as a possible destruction pathway of HC_3_N and a possible formation route of HCCN both in extraterrestrial environments and in the upper atmosphere of Titan. In particular, since both HC_3_N and HCCN are present in IRC+10216, we propose to search in this environment for also OCCCN (cyanoketyl), which is the other main product of the title reaction. We remind that nitriles are key intermediates in the formation of species with biological potential, such as nucleobases and amino acids.

We have also characterized the reaction dynamics of excited atomic oxygen, O(^1^D), with HC_3_N. It is interesting that this reaction leads to the same two product channels, with very similar branching fractions, as observed for the O(^3^P) reaction. However, while in the O(^3^P) reaction very efficient ISC in the entrance channel controls the reaction outcome producing electronically excited ^1^HCCN, in the O(^1^D) reaction, the same ^1^HCCN is formed adiabatically on the singlet PES. We wish to emphasize that ultimately, even in collision-less environments, the cyanomethylene product will be, from both reactions, in the ground electronic state, ^3^HCCN, because of spontaneous decay (or collisional quenching in dense environments) of ^1^HCCN.

The key intermediate in the global triplet/singlet PES of the title reactions is cyanoketene (COCHCN), which is the most stable singlet intermediate in the overall PES. It can be accessed via ISC from the O(^3^P) reaction or directly from the O(^1^D) reaction. Recent studies have suggested a possible role of O(^1^D) in comet^37,38^ and interstellar ice reactions.^39^ An intriguing reason for being interested in the reaction O(^1^D) + HC_3_N is that reactions of O(^1^D) with organic molecules present in interstellar ices can contribute to the formation of not only oxygenated organic molecules by surface stabilization of the most bound intermediates (such as cyanoketene in the title reactions), but also other oxygenated organic molecules with some loss of hydrogen on the surface.

Finally, the results of this study are expected to also be useful for improving not only astrochemical models but also combustion models involving the oxidation of cyanoacetylene, that is, combustion models of nitrogen-containing fuels.

## Supporting Information Available

Detailed triplet and singlet potential energy surfaces (PESs) for the O(^3^P) + HC_3_N and O(^1^D) + HC_3_N reactions, respectively, and Cartesian coordinates of the optimized geometries for the triplet PES and the singlet PES (PDF)
