# Supplementary material for: Reactions O(3P, 1D) + HCCCN(X1Σ+) (Cyanoacetylene): Crossed-Beam and Theoretical Studies and Implications for the Chemistry of Extraterrestrial Environments
Source: J Phys Chem A. 2023 Jan 13;127(3):685–703. doi: 10.1021/acs.jpca.2c07708 (PMC9884085; doi:10.1021/acs.jpca.2c07708)
Supplement: Supplementary file 1 — jp2c07708_si_001.pdf [file jp2c07708_si_001.pdf]

Supporting Information (SI) for the paper

Reactions $O(^3P, ^1D) + HCCCN(X^1\Sigma^+)$ (Cyanoacetylene): Crossed-Beam and Theoretical Studies and Implications for the Chemistry of Extraterrestrial Environments

Pengxiao Liang,[a] Emilia V. F. de Aragão,[a,b] Giacomo Pannacci,[a] Gianmarco Vanuzzo,[a] Andrea Giustini,[a] Demian Marchione,[a] Pedro Recio,[a,] Francesco Ferlin,[a] Domenico Stranges,[c] Noelia Faginas Lago,[a] Marzio Rosi,[d] Piergiorgio Casavecchia,[a]* and Nadia Balucani[a]*

[a]*Dipartimento di Chimica, Biologia e Biotecnologie, Università degli Studi di Perugia, Perugia, Italy*
[b]*Master-Tec srl, Via Sicilia 41, 06128 Perugia, Italy*
[c]*Dipartimento di Chimica, Università degli Studi La Sapienza, Roma 00185, Italy*
[d]*Dipartimento di Ingegneria Civile e Ambientale, Università degli Studi di Perugia, Perugia, Italy*

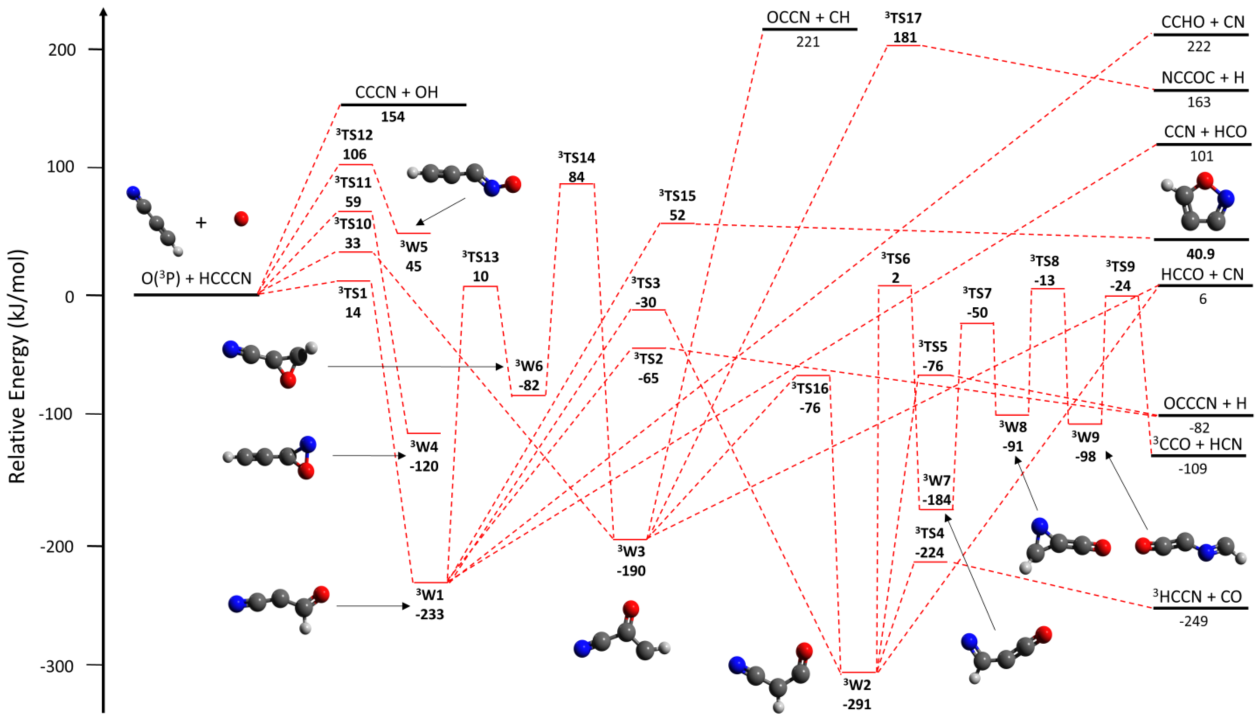

(a)

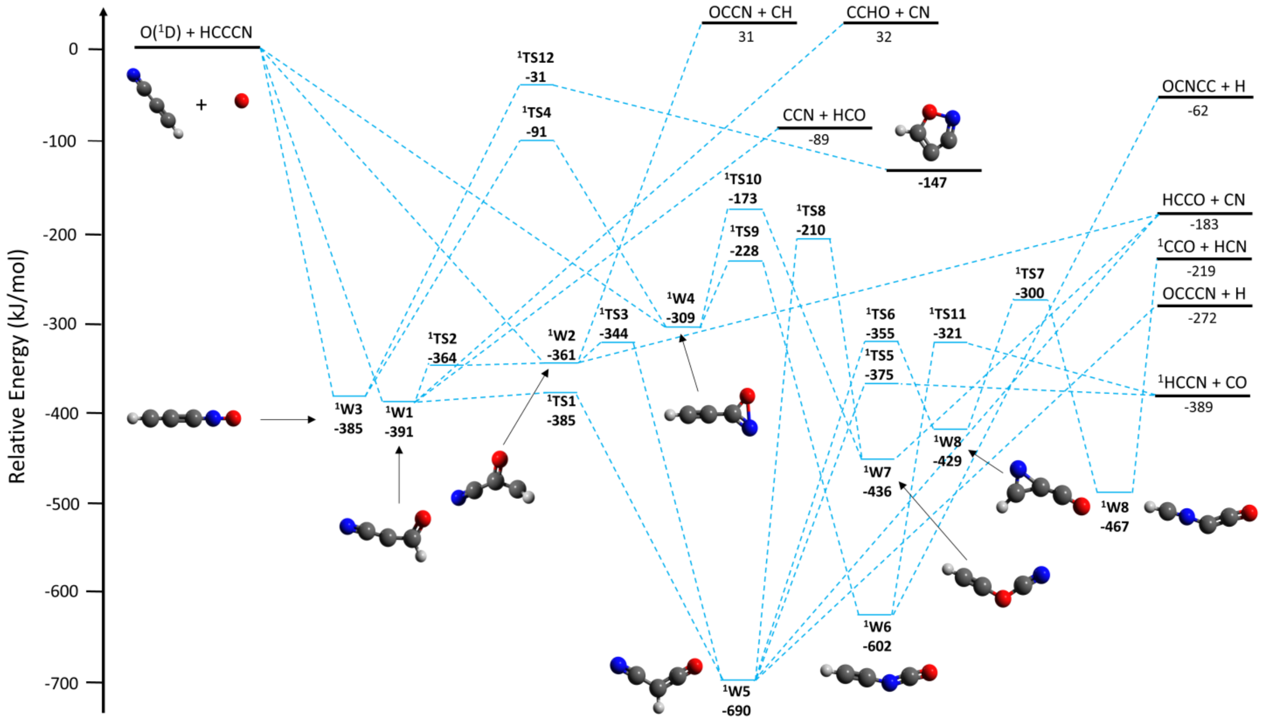

(b)

Figure S1. Potential energy surface arising from (a) the reaction of HC_3N with $O(^3P)$ and (b) the reaction of HC_3N with $O(^1D)$, calculated at the CCSD(T)/aug-cc-pVTZ//B3LYP/aug-cc-pVTZ level of theory. The reactants are taken as reference for the zero-energy level. For the sake of simplicity, a few endothermic pathways have been neglected in both PESs. In particular, all pathways starting from $^3W4$ and $^3W5$ have been disregarded due to their endothermicity and high entrance barriers leading to the adducts, therefore

totally inaccessible at the collision energy of 31.1 kJ/mol. In (a) the reported energy for the saddle point is below the energy of ^{1}W2, and this is explained by the fact that the zero-point energy correction is larger for the adduct than for the saddle point. In contrast, the electronic energy at B3LYP/aug-cc-pVTZ level for these two structures shows that the transition state is located above the adduct.

Cartesian coordinates (in Å) of the optimized geometries

TRIPLET PES

^{3}W1

C	-1.23823200	0.37121700	0.00005900
C	0.07757600	-0.18237500	0.00061000
C	1.38531800	-0.04430800	0.00030900
N	2.57196000	-0.00651800	-0.00046100
H	-1.26451500	1.47628200	-0.00009100
O	-2.26089700	-0.28723200	-0.00031900

^{3}W2

C	-1.34659900	0.34729700	0.00000000
C	0.00000000	0.91812100	0.00000000
C	1.13871000	0.12083700	0.00000000
N	2.07507300	-0.56862100	0.00000000
H	0.10768000	1.99227800	0.00000000
O	-1.67323100	-0.79118300	0.00000000

^{3}W3

C	1.20610500	1.16481500	0.00002600
C	0.47795700	-0.04054100	0.00016400
C	-0.98386700	0.04134700	0.00002200
N	-2.13248900	0.10324000	-0.00008100
H	2.27381200	1.33489900	-0.00008300
O	1.05655400	-1.13141300	-0.00007800

^{3}W4

C	2.24967800	-0.00310900	-0.06697600
C	1.03956600	0.02230800	0.01569100
C	-0.29615100	0.07568800	0.30064000
N	-1.48169800	0.72158000	-0.10785600
H	3.30630900	-0.02814400	-0.16869000
O	-1.36162300	-0.69903000	-0.07155600

^{3}W5

C	-2.48943200	-0.05028800	0.06691700
C	-1.27567400	0.03914100	-0.02928200
C	0.03325400	0.32814000	-0.11416200
N	1.12075200	-0.35485700	-0.07892700
H	-3.54205500	-0.16387000	0.14991700
O	2.26098700	0.09323900	0.10771700

^{3}W6

C	-1.52483300	-0.62791900	-0.05294200
C	-0.22919500	-0.09744200	-0.30269100
C	1.11204700	-0.04579100	-0.01495400
N	2.26955300	-0.02350600	0.10286500
H	-1.96288100	-1.18295100	0.77025600
O	-1.25901400	0.74680100	0.09165100

^{3}TS1

C	-1.07115500	1.00774200	0.00000000
C	0.05246200	0.55923200	0.00000000
C	1.30448600	0.01671500	0.00000000
N	2.37064900	-0.43003300	0.00000000
H	-1.99526600	1.53217900	0.00000000
O	-2.03925400	-1.00301000	0.00000000

^{3}TS2

C	-1.16041500	-0.00537600	-0.00000300
C	0.07383600	-0.43065100	0.00000000
C	1.36640000	-0.08407700	0.00000100
N	2.51550000	0.14652200	0.00000000
H	-0.67977100	1.90464800	0.00000100
O	-2.32595700	0.02378900	0.00000100

^{3}TS3

C	-1.29191500	0.33270700	-0.11374200
C	0.06680800	0.56048200	-0.08401100
C	1.31439800	0.00302300	-0.02141100
N	2.42921600	-0.33557300	0.04140900

H	-0.84216600	1.37815600	0.60751900
O	-2.08726200	-0.55080300	0.05220100

^{3}TS4

C	-1.67693700	0.37808500	0.00028300
C	0.42377900	1.07635200	-0.00007600
C	1.33719200	0.11241400	-0.00001200
N	2.06783500	-0.81796000	0.00005900
H	0.38427800	2.14648100	-0.00033000
O	-1.92041600	-0.72773400	-0.00015600

^{3}TS5

C	-1.17196300	-0.05345000	-0.00000100
C	0.04507000	0.39209200	-0.00001000
C	1.32245600	-0.02553400	-0.00000100
N	2.45428700	-0.32466100	0.00000400
H	0.15112500	2.51998300	0.00001600
O	-2.31306300	-0.26575000	0.00000400

^{3}TS6

C	-1.33826100	-1.07119200	0.00000000
C	-0.30361200	-0.42108800	-0.00000100
C	1.04838600	-0.14868200	-0.00000300
N	2.18251300	0.06115700	0.00000200
H	-2.31181900	-1.49981300	0.00000500
O	-1.17560700	1.36468500	0.00000000

^{3}TS7

C	-1.07115500	1.00774200	0.00000000
C	0.05246200	0.55923200	0.00000000
C	1.30448600	0.01671500	0.00000000
N	2.37064900	-0.43003300	0.00000000
H	-1.99526600	1.53217900	0.00000000
O	-2.03925400	-1.00301000	0.00000000

^{3}TS8

C	2.42522700	-0.39019500	0.00000000
C	1.26511100	-0.03596700	0.00000000
C	0.00000000	0.39770800	0.00000000

N	-1.15158300	0.73004800	0.00000000
H	3.43904900	-0.70856500	0.00000000
O	-2.18999900	-0.52888200	0.00000000

^{3}TS9

C	1.45028600	-0.55341100	-0.14313300
C	0.11951700	-0.27044800	0.18643400
C	-1.18863200	-0.09771900	-0.00814700
N	-2.34732300	0.11233300	-0.04786100
H	2.20823800	-1.28215900	0.11465400
O	1.49200000	0.75316200	0.00118100

^{3}TS10

C	1.44825300	0.82538700	-0.10920300
C	0.31041600	0.03723600	-0.04683900
C	-1.07266400	0.03636100	-0.00262100
N	-2.23371400	0.06718900	0.01986500
O	1.20379300	-0.93394000	0.03758500
H	1.88962100	1.60729100	0.51224400

^{3}TS11

C	1.12588200	0.11542000	-0.00000400
C	0.32574600	1.19735000	0.00000100
C	-0.99162400	0.67639100	0.00001000
N	-1.20923700	-0.52793000	-0.00001400
H	2.20165100	0.02469300	-0.00003500
O	0.43787300	-1.03301800	0.00001200

^{3}TS12

C	-0.18871500	1.28875800	-0.00000100
C	-0.66608200	-0.00758900	-0.00000400
C	0.81061100	-0.17890300	-0.00000300
N	1.96772800	-0.45861700	0.00000700
H	-0.36079800	2.35648300	0.00001100
O	-1.64352300	-0.71997000	-0.00000100

^{3}TS13

C	-1.38343700	0.88094300	-0.00017600
C	-0.28183500	0.08466700	-0.00005700

C	1.12951800	0.04621000	0.00010400
N	2.28130900	0.05390200	0.00022900
H	-3.48276500	0.51286800	0.00035900
O	-1.15898400	-0.87013800	-0.00014800

SINGLET PES

^{1}W1

C	-1.34472900	0.43834200	-0.00139100
C	0.06117200	0.39059600	0.00357900
C	1.31658600	0.03403400	0.00069400
N	2.47406400	-0.23165500	-0.00165200
H	-1.84090700	1.41749500	-0.00545000
O	-1.95946400	-0.62171800	-0.00003500

^{1}W2

C	1.35849700	0.96377200	-0.14642900
C	0.39382000	0.01628800	0.00185500
C	-1.04399700	0.03365900	-0.00530600
N	-2.19321100	0.07630000	0.01825000
H	1.94468300	1.32770400	0.69118100
O	1.14473500	-0.99301500	0.01004400

^{1}W3

C	-2.55629900	0.00020700	-0.00000200
C	-1.35104700	0.00025800	0.00000100
C	0.00119600	0.00022300	0.00000200
N	1.16362200	0.00004800	0.00000100
H	-3.61796400	-0.00258400	0.00000200
O	2.36368900	-0.00023600	-0.00000100

^{1}W4

C	2.19981700	-0.02021700	-0.00008400
C	1.00232400	0.02817800	0.00012900
C	-0.40256500	0.07745200	0.00026100
N	-1.34620500	0.90705100	-0.00011800
H	3.26198900	-0.06333900	-0.00032400
O	-1.32950100	-0.84981200	-0.00008500

^{1}W5

C	-1.12263300	0.08480200	-0.00003400
C	0.03196200	0.73197000	-0.00061200
C	1.26802300	0.04909200	-0.00023300
N	2.29264400	-0.48109200	0.00025900
O	-2.13855000	-0.45508600	0.00014500
H	-0.00422100	1.81314100	0.00230100

^{1}W6

C	-2.44319100	-0.15541600	-0.00000100
C	-1.25974000	0.04342200	0.00000400
C	1.17026800	0.01525800	0.00001400
H	-3.48500800	-0.35246100	-0.00000900
N	0.01682000	0.33799400	-0.00000600
O	2.32040500	-0.17913500	-0.00000600

^{1}W7

C	-2.17860500	-0.39211700	0.00000500
C	-1.09421600	0.10124200	0.00003300
C	1.16519800	0.03630600	0.00007300
N	2.17884500	-0.50928900	-0.00003500
H	-3.13658200	-0.84775300	-0.00010500
O	0.06630000	0.74252300	-0.00004000

^{1}TS1

C	-1.18887800	0.32444400	0.21234800
C	0.06281900	0.49901400	-0.43812300
C	1.27607800	0.03972400	-0.06987700
N	2.37232100	-0.30530600	0.16820700
H	-1.36067100	1.14489800	0.95111700
O	-2.01821000	-0.52335600	-0.04433200

^{1}TS2

C	1.37358900	0.74845400	0.00011000
C	0.27479400	0.11237000	-0.00032900
C	-1.11938200	0.06015900	-0.00001300
N	-2.27092100	0.00087100	0.00009700
H	2.29638000	1.28266100	0.00015900

O	1.30325800	-0.85183200	0.00006900

[1]TS3

C	0.87306000	1.32279400	-0.15731100
C	0.57213500	-0.05096900	-0.00486400
C	-0.92093200	-0.11537800	-0.02648700
N	-2.07279400	-0.10143000	0.02062300
H	0.92096200	1.90620300	0.76893000
O	1.30537700	-1.01685900	0.02733600

[1]TS4

C	2.38846400	0.18753100	0.00019700
C	1.19477800	-0.04897800	0.00026000
C	-0.09899600	-0.31911700	-0.00050800
N	-1.29787100	-0.60044700	0.00017900
H	3.43163900	0.39296500	-0.00096800
O	-1.90650200	0.61169300	0.00000300

[1]TS5

C	1.64640700	0.04312700	0.39988100
C	-0.64566600	1.06386100	-0.22375000
C	-1.54460500	0.07395800	-0.01630200
N	-2.29953100	-0.82479300	0.03617000
H	-0.50179500	1.88909900	0.46118500
O	2.48271200	-0.40015300	-0.20916900

[1]TS6

C	0.72185400	0.13876800	0.00000500
C	1.60284400	-0.75865300	-0.00000700
C	-0.75358300	-0.16139700	0.00000200
N	-1.82893600	-0.59782500	-0.00000200
H	2.00832900	-1.74439200	0.00001900
O	0.17094200	1.32710700	-0.00000100

[1]TS7

C	-2.06043600	-0.17428000	0.00009600
C	-0.85308400	-0.22104100	0.00004000
C	0.58729500	-0.01336400	-0.00002800
N	0.67136300	1.27713600	-0.00002700

H	-3.12443600	-0.17142700	0.00014700
O	1.54778000	-0.78955200	-0.00007600

^{1}TS8

C	-1.16041500	-0.00537600	-0.00000300
C	0.07383600	-0.43065100	0.00000000
C	1.36640000	-0.08407700	0.00000100
N	2.51550000	0.14652200	0.00000000
H	-0.67977100	1.90464800	0.00000100
O	-2.32595700	0.02378900	0.00000100

^{1}TS9

C	2.53798900	-0.28819600	0.15698500
C	1.30801900	0.11562600	-0.02463800
C	-1.37690300	-0.43412700	-0.00567000
N	0.20846100	0.62192100	-0.01359900
H	3.19379400	-0.48843200	-0.68276800
O	-2.43345600	-0.02810400	0.00223800

^{1}TS10

C	-1.20337300	0.66347300	-0.04161800
C	-1.24874400	-0.60630500	-0.07318100
C	0.02525300	-0.92318100	0.16016600
N	1.16890000	-0.46491600	-0.09335900
H	-1.49560600	1.66385800	0.19536800
O	0.98431100	0.84832900	0.02324200
